# Experiences of LGBTQ+ graduate students in research-focused doctoral programs: a scoping review

**DOI:** 10.3389/feduc.2024.1472113

**Published:** 2024-10-20

**Authors:** Carrie Baldwin-SoRelle, David A. McDonald

**Affiliations:** 1 Health Sciences Library, University of North Carolina at Chapel Hill, Chapel Hill, NC, United States; 2 Office of Graduate Education, School of Medicine, University of North Carolina at Chapel Hill, Chapel Hill, NC, United States

**Keywords:** LGBTQ, graduate students, SGM, intersectionality, belonging, mental health, professionalism, mentorship

## Abstract

Students of sexual and gender minority (SGM) identities have long been underserved in higher education, and the limited research thus far has focused on undergraduates. There is a large gap in understanding the outcomes and experiences of LGBTQ+ graduate students, particularly in STEM. We undertook the first scoping review to examine the available literature on LGBTQ+ student experiences in research-focused doctoral programs. A scoping review methodology was utilized to compile a broad set of publications for a narrative review of emergent themes. A comprehensive search of 5 bibliographic databases yielded 1,971 unique studies, which were screened by two independent reviewers for data on LGBTQ+ doctoral students in non-clinical fields. Eighty-two publications were included in the analysis, over half of which were published in the past 5 years. Thirteen themes emerged from analyzing the included publications. LGBTQ+ ientities can continue evolving during graduate school, and some students incorporated SGM identities in their research (“mesearch”). Though students expected academia to be welcoming, many encountered repeated anti-LGBTQ+ bias that impacted their perceived safety for coming out. Nearly half of the studies mentioned intersectionality with other marginalized identities, including race/ethnicity, religion, disability, and others. Based on the information presented, we outline recommendations for practitioners to improve doctoral education, such as preparing teaching assistants to manage discriminatory classroom conduct.

## 1 Introduction

People of lesbian, gay, bisexual, transgender, queer, and other identities (LGBTQ+) have experienced increased social support in the United States over the past few decades (Flores, 2014), but there has been a marked increase in anti-LGBTQ+ legislation in recent years (ACLU, 2024). There are currently no federal data sets about educational attainment based on sexual orientation or gender identity (SOGI). Gay and lesbian individuals are more likely than heterosexuals to hold bachelor’s or advanced degrees (Mittleman, 2022). Despite this, diversity, equity, and inclusion (DEI) efforts in higher education have focused more on race, ethnicity, binary gender, socioeconomic status, and disability, without a similar level of attention to LGBTQ+ identities. Similarly, guidance from the National Institutes of Health on diversity does not include SOGI in definitions of “underrepresented” (https://grants.nih.gov/grants/guide/notice-files/NOT-OD-20-031.html). These definitions note that women, for example, are underrepresented at the faculty level, particularly among senior leadership roles, but LGBTQ+ individuals are absent from the NIH’s definitions despite similar evidence of historical exclusion and minoritization.

The first large-scale studies of campus climate for LGBTQ+ people started in the 2000s (Rankin, 2003). Most of the research since then has focused on undergraduate populations (Cech and Waidzunas, 2011; Kilgo et al., 2019; Jennings et al., 2020), faculty (LaSala et al., 2008; Bilimoria and Stewart, 2009), and the STEM workforce (Yoder and Mattheis, 2016; Cech and Pham, 2017). The National Science Foundation has only recently piloted questions about SOGI in their Survey of Earned Doctorates. As a result, little is known about the experiences of LGBTQ+ graduate students in doctoral programs, a key developmental point between undergraduate studies and careers in academia or the broader scientific workforce. Furthermore, fields of science, technology, engineering, and math (STEM) have been slow to consistently include SOGI in educational research, likely due to cultures of cisheteronormativity (Miller et al., 2021), defined as the social enforcement that being cisgender and heterosexual are “normal” and the default. As a result, there is limited information on the successes and challenges that LGBTQ+ students face when pursuing advanced degrees in STEM.

To begin to address these gaps, we examined the extant literature for information on the experiences of doctoral students of sexual and gender minority (SGM) identities. The goals were to compile a broad view of the data and perspectives that have been published, and to propose action steps for improving graduate education environments. A scoping review methodology was employed to leverage rigorous literature searching to better inform a narrative review using emergent themes. Since there have been a relatively small number of studies on this topic in STEM fields, we included any research-focused academic field that was not clinical in nature. We included data from a wide variety of methodologies and theoretical frameworks. Due to the limited number of peer-reviewed research publications, we included additional types of publications (ex. dissertations, book chapters, and editorials). Again, the goal of this work is to elevate the variety of experiences of LGBTQ+ doctoral students beyond the few studies published in a particular field, not to quantify the experiences or combine data sets; indeed, there are too few studies and data sets to attempt meta-analysis. Also, since identities are not discrete elements, we analyzed the included publications for narratives about SOGI intersecting with other identities (Crenshaw, 1989).

A note on terminology: there is ongoing debate about inclusive language to encompass the wide variety of human SOGIs. For this publication, we chose LGBTQ+ as a general umbrella term representing all minoritized SOGIs. We acknowledge the limitations of this choice, and we in no way want to exclude intersex, asexual, agender, aromantic, two-spirit, nonbinary, or other identities from this discussion. Sexual and gender minority (SGM) will be used interchangeably with LGBTQ+ in this text. SGM is used more widely in the recent education literature, but it is not as well recognized in popular culture as LGBTQ+.

## 2 Methods

**FIGURE 1:**
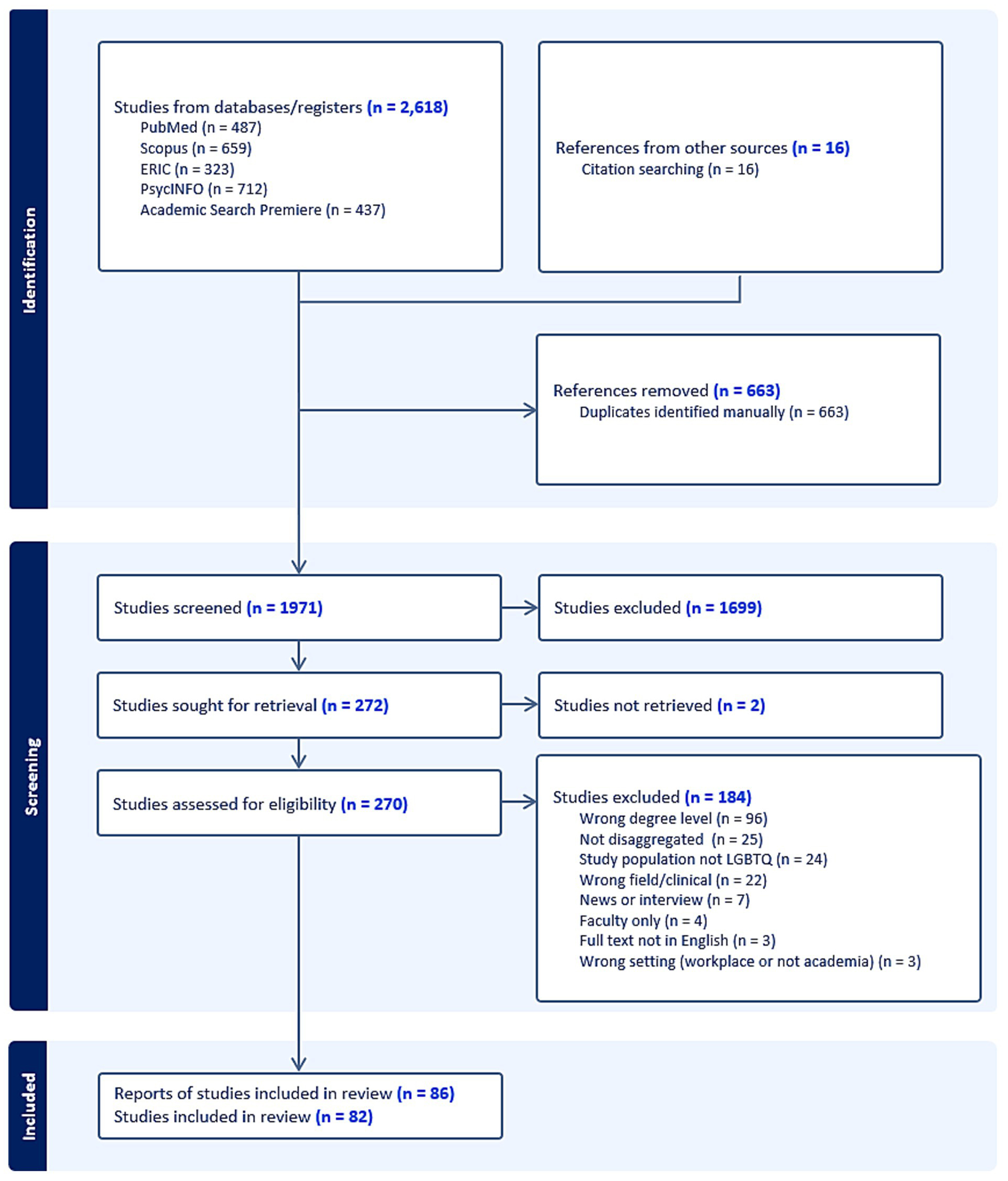
PRISMA Flow diagram of search and screening process. Studies were first identified across five databases/registers (*n* = 2,618), which were then de-duplicated. The remaining 1,971 studies were screened for inclusion and exclusion criteria. A total of 86 reports fit the criteria, representing 82 studies. Reports vs. studies: reports are total number of items that came through in the search; the number of included studies reflects that some items that were multiple chapters in the same book were combined.

The authors designed a comprehensive search using an extensive range of indexed terms and keywords based on the concepts of SGM and graduate school (Figure 1). CBS, a health sciences librarian, searched PubMed, Scopus, Education Resources Information Center (ERIC), PsycInfo, and Academic Search Premier to identify literature across health, social, and natural sciences disciplines, yielding 2,618 studies in total. All databases were searched on June 28, 2023, without date restrictions. The full search strategies for each database are available in Supplementary Figure S1. The team conducted backwards citation searching on all included studies.

After deduplication, two independent reviewers screened 1,971 studies for relevance at the title and abstract stage, and then retrieved the full text of 270 studies. Studies were evaluated for the following inclusion criteria: they (1) discussed doctoral students; (2) in non-clinical research fields, (3) who have SGM identities. Graduate students in clinical programs (e.g., psychology, social work) often have experiences as learners and as client-serving practitioners, which is a sufficiently different training environment that they were excluded from this review. A preliminary analysis of the publications found that articles from clinical fields, such as psychology and social work, studied a mix of subjects (LGBTQ+ students and LGBTQ+ clients) that could be difficult to disentangle, so these fields were excluded from this project. Studies were also excluded if they did not specify or disaggregate by degree level; did not consider SGM graduate students; included only faculty; had a setting outside higher education; were written in languages other than English; or were news or interviews. A total of 82 studies were included in the analysis (Supplementary Table S1). We followed the Preferred Reporting Items for Systematic Reviews and Meta-Analyses extension for Scoping Reviews (PRISMA-ScR) in reporting this review (Tricco et al., 2018).

The included publications were then analyzed for: type of publication, field of study, year published, methodology, theoretical framework, intersectional demographics, and major findings. Data and results from these publications were examined for common emergent themes, which served as the basis for the narrative review presented below. This review qualitatively highlights the variety of perspectives and experiences in the literature. In this way, these publications are intended to be illustrative rather than a definitive and finite list.

### POSITIONALITY:

The lead author (CBS) identifies as a white cisgender lesbian. The last author (DAM) identifies as an able-bodied gay white cisgender man who was the first in his family to attend a doctoral program. These identities grant personal connections with this project, but also potential privileges and biases.

## 3 Results

### 3.1 Study characteristics

**FIGURE 2:**
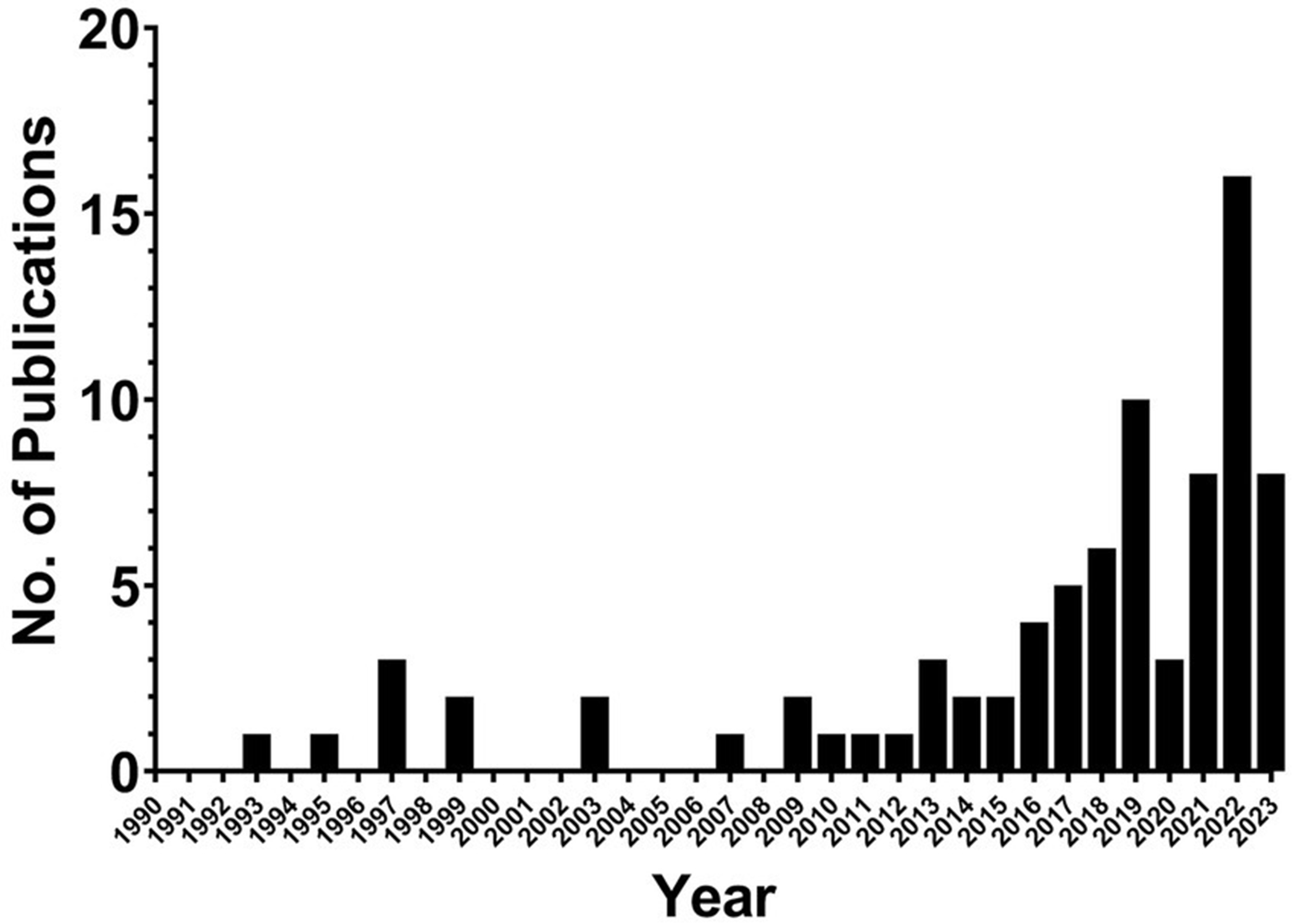
Publication frequency of included studies. number of publications per year included in the scoping review.

Of the 82 texts included in this review, over half were published during or after 2019 (in the past 5 years). As shown in Figure 2, the earliest study included was published in 1993, and until 2016 an average of 1.7 texts per year about LGBTQ+ doctoral students were published. Starting in 2016, the number of texts published each year started to increase, peaking in 2022 with 16 publications.

Forty-eight of the texts studied (59%) were peer-reviewed articles from academic journals. The remaining items were 17 books or book chapters (21%), 10 dissertations (12%), 4 conference papers (5%), 2 editorials (2%), and 1 society report (1%). The majority of texts included information about experiences in the United States (91%). Other countries included were the United Kingdom, Mexico, Canada, New Zealand, Australia, and Chile.

Of the academic fields reflected in these works, the social sciences predominated with representation in 41% of the texts. Other fields were represented at similar but slightly lower levels: humanities and arts in 33% of the texts, STEM in 32%, and education in 28%. Within STEM, physical sciences were the highest (in 17% of texts), followed by life sciences (13%), engineering (10%), and computational fields (9%). Across all of the publications, 15% focused solely on STEM fields, 13% included STEM fields along with non-STEM fields, and the remaining 72% included no identifiable students from STEM fields. Academic disciplines were not specified in 13% of the items included (though it was clear from the texts that they were doctoral students), often to protect the identity of the participants. In looking at the numbers of participants in each study (68 of the publications provided total numbers), 72% included five or fewer individuals and 50% were from the perspective of a single individual.

The methodology used most across the texts (in 41%) was personal reflection and opinion in which LGBTQ+ authors recalled their individual experiences in their doctoral programs. Relatedly, eight additional studies employed autoethnography as a qualitative method to examine their experiences. Surveys were used in 37% of the texts, interviews in 32%, and focus groups in 6%. Though the included studies with STEM students trended toward use of surveys more often and opinion/reflection less often, this pattern was not statistically significant (Chi-square, *p* = 0.0602). The remaining texts utilized various other approaches: narrative review, text analysis, panel discussion, observations, and performative writing. Notably, two of the earlier publications included were case reports by therapists working with gay graduate student clients (Gould, 1999; Levounis, 2003). Four texts described an intervention with students that was studied, all of which were approaches to building community (Burford, 2017; Nadal, 2019; Al-Saleh and Noterman, 2021; Bakka et al., 2021).

Twenty-nine publications indicated using a specific theoretical framework. The most prevalent frameworks were queer theory, feminist theory, minority stress frameworks, and intersectionality. Other frameworks mentioned include Pedagogy of the Oppressed, hope theory, cultural capital, jotería studies, and genre theory. A complete list of the theoretical frameworks, publication type, methodology, fields, and number of participants for each publication are shown in Supplementary Table S1.

In examining the approaches used, 20 of the survey-based studies, though they met our inclusion criteria for LGBTQ+ doctoral students, had samples too small to allow for robust analysis. In some studies, transgender (trans) and nonbinary students’ data were combined with the data for women-identified students. In others, the data from graduate students of minoritized SOGIs were excluded from analysis altogether. In still others, LGBTQ+ graduate students and undergraduates were combined into a single group. Besides this variety of data strategies, many studies focused on qualitative methodologies. Since meta-analysis across the included publications was not possible, we set out to summarize, compare, and contrast the results in the following sections of this review.

### 3.2 Themes

After assembling this collection of 82 publications, the findings were examined to understand a breadth of experiences SGM doctoral students have had across academic fields. The topics that emerged were grouped into 13 themes by the authors (summarized in Table 1 and fully described in Supplementary Table S2). Since the purpose here is not to focus on the quantitative results (i.e., proposing that more frequent themes are more important), the themes are presented in a narrative order chosen by the authors for easier readability.

Before further examining the themes, it is important to point out that LGBTQ+ identities are not a monolith; the letters of that acronym not interchangeable, and not all people of a particular minoritized SOGI share the same experiences. Instead, this article highlights the variety of perspectives found in the published literature.

#### 3.2.1 Identities under formation

A prevalent theme mentioned in 28 of the articles in this review is that LGBTQ+ students’ identities may still be forming when they enter a doctoral program. In that way, graduate school was a place of becoming, self-discovery, and new experiences (Bailey and Miller, 2015; Lee, 2017; Crawley, 2021; Cross et al., 2022). SGM students in graduate school may explore different labels, evolve, and transition (Smith, 1995; Mintz and Rothblum, 1997; Resides, 1997; Cortez, 2013; Burford, 2017; Nowakowski and Sumerau, 2017; Ortis, 2018; Phillips, 2018; Beemyn, 2019; Bhattar, 2019; Singh and Mathews, 2019; Gilliam and Swanson, 2020).

For some LGBTQ+ students, graduate school was the first time they could connect with other people of similar SOGIs or literature relevant to their identities (Bakka et al., 2021; Crawley, 2021; Duran et al., 2022). Students represented in these publications mentioned that their LGBTQ+ identities can feel more or less salient in different situations, and they find themselves combatting internalized homophobia, biphobia, and transphobia (where individuals of a minoritized identity demonstrate a bias against that identity). These students may experience changes in their SOGI and academic identities simultaneously (Pierce, 2003; Barbier, 2007; Cisneros et al., 2022; Platt et al., 2022). Platt et al. (2022) conducted a survey of graduate students across natural and social science fields and found that SGM students reported identifying as scientists less strongly compared to heterosexual students (Platt et al., 2022). Two studies also modeled the interactions between researcher identity and gender/sexuality in graduate students (Satterfield et al., 2019; Bahnson et al., 2021).

#### 3.2.2 Intersectional identities

Table 2 includes a summary of the 36 publications that described graduate students’ intersectional identities with SOGI. Race and ethnicity appeared the most frequently, followed by socioeconomic status/class, disability, international status, and religion. LGBTQ+ graduate students with additional marginalized identities often reported feeling like their multiple identities competed with each other, and there were times when SOGI did not feel as salient or central as other identities (Resides, 1997; Mehra, 2016; Means et al., 2017; Nowakowski and Sumerau, 2017; Cross et al., 2022; Reggiani et al., 2023). Students may find that they have to focus on one marginalized identity at a time for their own self-preservation (Ortis, 2018; Cisneros et al., 2022; Cross et al., 2022). They can feel a disconnect from others who have a single marginalized identity (Ortis, 2018). As stated in Bailey and Miller, “when all the queers are white and all the Black folks are straight, what is a Black queer woman to do?” (Bailey and Miller, 2015). Graduate students with multiple marginalized identities can develop outsider feelings due to rejections from multiple groups (Cortez, 2013; Smith, 2014; Mehra, 2016; Lee, 2017; Means et al., 2017; Bhattar, 2019; Coloma, 2020; Duran et al., 2022; Martinez, 2023). Even spaces dedicated to support LGBTQ+ students can feel exclusionary if intersectional identities are not included (Lee, 2017).

These students can face multiple types of discrimination in addition to homophobia, transphobia, and cisheterosexism (Misawa, 2009; Cortez, 2013; Bailey and Miller, 2015; Handy, 2016; Mehra, 2016; Lee, 2017; Ortis, 2018; Bhattar, 2019; Singh and Mathews, 2019; Cisneros et al., 2022; Cross et al., 2022; Whitley et al., 2022; El Kurd and Hummel, 2023; Martinez, 2023; Reggiani et al., 2023). For example, Glover (2017) specifically mentions experiences of “misogynoir,” a gendered form of racism that targets Black women (Glover, 2017). Furthermore, microaggressions were associated with higher rates of anxiety among LGBQ+ (trans identities omitted) people of color compared to white heterosexual men (Boyle et al., 2022).

Students with multiple marginalized identities can have layered and complicated experiences of academia based on their identity groups and cultures. As discussed earlier in this article, academia can reinforce stereotypically masculine traits and behaviors, and this can compound with masculinities and patriarchies in, for example, Black, Latinx, Asian-Indian, and religious cultures (Mehra, 2016; Means et al., 2017; Cross et al., 2022). Along those lines, Handy’s (2016) study includes a “Black gay male who is often misperceived as a straight and ‘angry’ male” (Handy, 2016). In Ortis (2018) work as well, a student found that their LGBTQ+ and polyamorous identities reinforced negative racial stereotypes (Ortis, 2018). Students encountered a variety of people in academic spaces whose identities and cultures defined SOGI differently than Western cultures (Mehra, 2016). Such cultural heterogeneity can even complicate the gendered expectations and beauty standards that graduate students experience in their doctoral programs (Lyle et al., 1999). For students seeking religious community, they may find it challenging to find groups that are inclusive of LGBTQ+ people (Resides, 1997).

Graduate students in these publications relied on their connections with other people of intersectional identities to persist in their doctoral programs, both in and outside of academia (Bailey and Miller, 2015; Glover, 2017; Means et al., 2017; Nadal, 2019; Singh and Mathews, 2019; Coloma, 2020; Sokolowski, 2020; Duran et al., 2022). Beyond the paucity of LGBTQ+ faculty in general, there are even fewer faculty with additional marginalized identities (Cortez, 2013; Means et al., 2017; Nadal, 2019; Singh and Mathews, 2019; Wright-Mair and Marine, 2021; Reggiani et al., 2023). So graduate students may seek alliances with groups of non-LGBTQ+ minoritized students for support (Handy, 2016), though this may be hampered by the way those groups treat LGBTQ+ people and rivalries between minoritized groups for limited resources (Misawa, 2009). For the students in these publications who found acceptance of one marginalized identity, that can embolden them to claim others, such as a doctoral student who was supported after coming out as gay and went on to be more vocal about his Latinx identity (Cross et al., 2022). When there are multiple students with intersectional marginalized identities in a classroom, they may also feel more comfortable participating in discussions (Strings and Nasir, 2022).

#### 3.2.3 Motivations and constraints for starting graduate school

LGBTQ+ students shared motivations for why they wanted to pursue a doctoral degree in seven publications. They referenced desires to conduct research, work with faculty, and build a career, but there were also motivations specific to LGBTQ+ experiences. Students looked to academia to seek refuge (Levounis, 2003; Duran et al., 2022), especially if they had been disowned by their family (Glover, 2017). Ings writes of it this way: “For some queer students growing up, they recall the comparative safety of the school library or art room” (Ings, 2015).

In starting graduate school, multiple publications mentioned the financial stressors experienced by LGBTQ+ doctoral students. If their families are not supportive of their identities, students may be completely financially independent, and they may bear additional financial constraints in accessing medical treatments, hormones, or therapy services (Goldberg et al., 2022). Handy (2016) noted that a student chose their doctoral program based on affordability (Handy, 2016), and Glover (2017) mentioned that a student took out loans to enter a PhD program (Glover, 2017).

#### 3.2.4 Unmet expectations of graduate school

While SGM graduate students may have held specific expectations of academia when starting their doctoral programs, 14 studies detailed ways their actual experiences fell short. LGBTQ+ students may expect academia generally, or their field specifically, to be welcoming of diverse identities (Duran, 2021; Kawano, 2020; Misawa, 2009; Nowakowski and Sumerau, 2017; Ortis, 2018). Some are disappointed that not all their professors or peers are as affirming as they had hoped (Lee, 2017; Ortis, 2018; Samek and Donofrio, 2013). They may expect different levels of acceptance at Primarily White Institutions (PWIs), Historically Black Colleges and Universities (HBCUs), and universities with religious ties (Duran et al., 2022; Glover, 2017; Singh and Mathews, 2019). Students may assume that institutions in the American Midwest would be more hospitable compared to the South (Glover, 2017), or in the US overall compared to other countries (Mehra, 2016). However, many of these students’ actual experiences are not as positive as they had hoped, and they still faced bias and discrimination. In that way, these SGM graduate students feel that universities do not always live up to the image of diversity and acceptance that they project (Cortez, 2013; Hinchey and Kimmel, 2000; Reggiani et al., 2023). Conversely, their experiences can also be better than they might have feared, such as a student who was pleased to find that a religious university hosted a thriving LGBTQ+ student group in the chapel basement (Duran et al., 2022).

#### 3.2.5 Coming out is a decision made multiple times per day

As SGM students begin to navigate academic spaces, they made choices about whether and how to share their LGBTQ+ identities with those around them (“coming out”), and this was one of the most prevalent themes in this study (in 45/82 publications). Reggiani et al. (2023) and Smith (1995) both highlight that coming out is not a single event; rather, these students re-evaluate their outness in different environments, sometimes on a daily or even hourly basis (Smith, 1995; Reggiani et al., 2023).

In the arc of graduate school, students start making these decisions when they prepare their application materials for doctoral programs. They may receive advice from faculty to remove LGBTQ+ content from their curriculum vitae (Beemyn, 2019; Goldberg et al., 2022), or they may strategically include LGBTQ+ content as a litmus test for how accepting a program is (Sokolowski, 2020). When deciding where to apply to graduate school, aspiring doctoral students see the trade-off between prioritizing their professional research goals and finding a supportive environment (Sokolowski, 2020). Students in the US may focus on programs in regions of the country that are more accepting (Mintz and Rothblum, 1997; Sokolowski, 2020; Duran et al., 2022).

LGBTQ+ graduate students are actively looking for signals of danger or safety in their learning environments. Placards for LGBTQ+ inclusivity training (Sokolowski, 2020; Cross et al., 2022) and gender-neutral language (Knutson et al., 2022) are seen positively. On the other hand, students may decide to remain closeted if they perceived the presence of people with conservative political beliefs (Atherton et al., 2016; Beemyn, 2019; Sokolowski, 2020), when faculty did not talk about their personal lives (Resides, 1997), when microaggressive comments were permitted (Sokolowski, 2020), or when SGMs did not fit into the prototypical model of a researcher (Cisneros et al., 2022). Cortez points out, too, that even diverse spaces are not always accepting or safe spaces (Cortez, 2013).

In graduate-level courses, SGM students may evaluate their safety based on the size of the class and whether queer topics or researchers are mentioned in the content (Turkowitz, 2012), which can even be seen as an invitation to come out through a class discussion or assignment (Ortis, 2018; Duran et al., 2022). Some students will purposefully bring up SOGI topics in class to evaluate how the instructor and other students react (Smith, 1995; Resides, 1997).

Within this theme, twelve publications mentioned a lack of LGBTQ+ faculty and staff as an impediment to coming out and to students’ overall success (Resides, 1997; Turkowitz, 2012; Cortez, 2013; Bailey and Miller, 2015; Ortis, 2018; English and Fenby-Hulse, 2019; Nadal, 2019; Sokolowski, 2020; Goldberg et al., 2021; Wright-Mair and Marine, 2021; Cross et al., 2022; Reggiani et al., 2023). Indeed, a survey of evolution scientists found that 33% of graduate student respondents identified as LGBQ+ (trans identities omitted), but only 14% of untenured faculty and 7% of tenured faculty identified as LGBQ+ (Rushworth et al., 2021). In some instances, there may be faculty in a department who are known to have an LGBTQ+ identity, but they are not publicly out and do not attempt to connect with LGBTQ+ students (Resides, 1997). Students also observed how others talk about SGM faculty in the department (Sokolowski, 2020). Out LGBTQ+ faculty can demonstrate to students that they would be welcome in that field (Goldberg et al., 2022), especially when students share additional, intersectional social identities with them (Cross et al., 2022).

Students’ decisions about whether to be out in graduate school may also stem from their past experiences in coming out. Some may be more reluctant if they were rejected by their family or friends (Resides, 1997; Gould, 1999; Levounis, 2003; Ortis, 2018; Coloma, 2020; Sokolowski, 2020) or if they endured harassment (Cross et al., 2022). Other students had positive past experiences in coming out and being supported, and that may embolden them to be out as a doctoral student (Lee, 2017; Ortis, 2018). Older LGBTQ+ graduate students in particular may feel more comfortable to be out in their program (Resides, 1997; Ullman et al., 2018).

Many of these publications described the negative consequences when LGBTQ+ graduate students came out in their program. Foremost among them were experiences of violence or fears of violence (Glover, 2017; Nowakowski and Sumerau, 2017; Ortis, 2018; Beemyn, 2019; Sokolowski, 2020). Students may avoid using gendered restrooms (Cortez, 2013; Knutson et al., 2022) or accessing resources specifically for LGBTQ+ people (Cross et al., 2022). Traveling outside of their home country for conferences or field work came with concerns of safety (English and Fenby-Hulse, 2019; Coloma, 2020; Reggiani et al., 2023). In addition, students experienced professional consequences, such as a loss of respect and feeling their perspectives were delegitimized (Ings, 2015; Atherton et al., 2016; Ortis, 2018; Beemyn, 2019; English and Fenby-Hulse, 2019; Cross et al., 2022). They worried that being out to their dissertation advisor would negatively impact their graduate training (Yitmen and Almusaed, 2022). In these environments, LGBTQ+ students often felt invisible and isolated, and they may have limited their social interactions in graduate school for self-protection (Resides, 1997; Hinchey and Kimmel, 2000; Turkowitz, 2012; Cross et al., 2022; Reggiani et al., 2023). They could choose to “pass” or “go stealth” to minimize their visibility, sometimes relying on privileges of their outward appearance or other social identities (Smith, 1995; Samek and Donofrio, 2013; Means et al., 2017; Beemyn, 2019; Cisneros et al., 2022; Cross et al., 2022; Maughan et al., 2022; Whitley et al., 2022; Reggiani et al., 2023).

There were also positive outcomes for SGM students who chose to come out. Many trans and nonbinary graduate students in one study were able to wear clothing matching their gender identity and to adopt a first name other than their birth name (Beemyn, 2019). Coming out could be met with curiosity, support, acceptance, and even an improvement in professional relationships (Sokolowski, 2020; Cross et al., 2022; Yitmen and Almusaed, 2022).

Depending on the environment, LGBTQ+ students may choose to be selectively out to those who seem supportive (Coda, 2023). After coming out, students may still not discuss their personal lives with others, or they may lie about their personal lives to certain people (Resides, 1997; Bailey and Miller, 2015; Sokolowski, 2020; Coda, 2023). Instead of discussing their identities, they may rely on small, visible accessories, such as rainbow bracelets or bumper stickers (Smith, 1995; Cross et al., 2022). Since coming out is not a one-time event, students may take time to evaluate different relationships and slowly, even over the course of years, come out to particular individuals and groups (Linley and Kilgo, 2018; Sokolowski, 2020; Reggiani et al., 2023). Phillips adds that it is helpful to have supportive individuals in one’s life when coming out to additional people (Phillips, 2018). A complication in this process occurs when students feel a disconnect between their identities and what they perceive as the accepted outward signals of being an out LGBTQ+ person, potentially based on stereotypes of physical appearance (Duran et al., 2022), butch vs. femme dichotomies (Lyle et al., 1999; Samek and Donofrio, 2013), or bisexual erasure (Ortis, 2018).

In spaces lacking LGBTQ+ visibility, graduate students may choose to come out as an act of defiance or advocacy (Cisneros et al., 2022; Cross et al., 2022; Reggiani et al., 2023). They can potentially protect and inspire younger LGBTQ+ students (Handy, 2016, Means et al., 2017, Ortis, 2018, Crawley, 2021, Cross et al., 2022). As mentioned by Wright-Mair and Marine (2021), “sometimes, when you come out, you become the most senior person of those identities at your department/institution” (Wright-Mair and Marine, 2021).

#### 3.2.6 Navigating teaching and management relationships

Related to outness, SGM students across 13 publications described how sharing their LGBTQ+ identities shaped their experiences as instructors and teaching assistants. Graduate students taught on a variety of topics, including a few directly related to their identities (Cortez, 2013; Bailey and Miller, 2015; Heffernan and Gutierez-Schmich, 2016; Mehra, 2016; Kawano, 2020). Some of these students came out to their classes to combat stereotypes (Cross et al., 2022) or to supplement the standard curriculum with information about intersectional identities (Glover, 2017). In one publication, an author described creating a “queer pedagogy” by embedding experiential learning opportunities in a class to subvert traditional academic hierarchies (Juhasz and Ma, 2009).

By contrast, other LGBTQ+ graduate students expressed fear of being outed to their students or altering the classroom dynamic (Barbier, 2007; Ortis, 2018). Some graduate students who were out to their classes experienced a wide range of negative reactions. The students they teach expressed apathy or were resistant to the subject matter (Bailey and Miller, 2015; Kawano, 2020). Their students also made discriminatory remarks (Heffernan and Gutierez-Schmich, 2016), retaliated with negative evaluations (Cortez, 2013), and sent intimidating emails (Doxbeck and Karalis Noel, 2023). LGBTQ+ graduate students felt unprepared and unsupported to handle these student behaviors, either when directed at them personally or directed toward other students in the class (Smith, 1995; Sokolowski, 2020).

Only one publication, a dissertation, mentioned the dynamics of an LGBTQ+ graduate student serving as a manager of employees. The student discussed their decision to not out themselves to their direct reports due to concerns about how it would alter the dynamics of the supervisory relationship (Ortis, 2018).

#### 3.2.7 Mesearch

In addition to mapping their identities into teaching, many LGBTQ+ doctoral students in 32 of these 82 studies wanted to engage in research that is connected to their personal identities or communities, so-called “me-search” or “mesearch.” There is an overrepresentation of non-STEM fields in the current literature – only 3 of the 32 studies with references to mesearch including STEM doctoral students.

For some students, mesearch starts in their graduate courses. This may be their first exposure to LGBTQ-inclusive literature and role models (Crawley, 2021), and they have opportunities to explore different queer identities and experiences (Cisneros et al., 2022). Students may notice an obvious lack of LGBTQ+ content in their courses and seek to fill those gaps themselves (Turkowitz, 2012).

Overall, this process can be empowering, helping fuel students’ research, community, and personal work (Cortez, 2013). Students can begin to confront internalized cisheterosexism (Gilliam and Swanson, 2020; Cisneros et al., 2022). They may feel called to mesearch topics (Duran, 2021). In sharing their research interests with the students they teach, they may form connections and serve as role models (Handy, 2016; Cross et al., 2022). As researchers, students may gain credibility in sharing identities with research subjects (Nowakowski and Sumerau, 2017; Gilliam and Swanson, 2020). Two studies described how students were able to remove barriers between researcher and subject, creating a shared endeavor and validating the participants (Nowakowski and Sumerau, 2017; Duran et al., 2022). When graduate students’ mesearch interests are encouraged and they have opportunities to build expertise, they can flourish (Samek and Donofrio, 2013; Lee, 2017; Ortis, 2018; Beemyn, 2019).

Multiple roadblocks impede SGM graduate students from conducting mesearch, though. In addition to a comparative lack of funding for these projects (Bhattar, 2019), students received little institutional support (Hinchey and Kimmel, 2000; Bhattar, 2019; Maughan et al., 2022). Mesearch projects were less valued by students’ departments, and they did not bring the same status as research not connected to personal identities (Smith, 1995; Pierce, 2003; Samek and Donofrio, 2013; Nowakowski and Sumerau, 2017; Wright-Mair and Marine, 2021). Faculty discouraged students from mesearch topics (Hinchey and Kimmel, 2000; Cisneros et al., 2022; Maughan et al., 2022; Whitley et al., 2022), or even harassed students for their interests (Resides, 1997; Hinchey and Kimmel, 2000; Misawa, 2009). For example, Hinchey and Kimmel (2000) describe a situation in which a faculty administrator repeatedly attempted to intimidate a doctoral student, and their SOGI-related dissertation title was omitted from the graduation program because it was deemed offensive (Hinchey and Kimmel, 2000).

A critique that students may receive about pursuing mesearch is that their personal connection prevents them from being objective (Nowakowski and Sumerau, 2017). Meanwhile, since these topics and populations are often understudied, graduate students can lack the mentorship needed to properly conduct mesearch projects (Nadal, 2019). Students may find that researchers with shared LGBTQ+ identities are more likely to compete rather than collaborate (Wright-Mair and Marine, 2021). The timelines for in-depth, human-centered research projects with these populations may not align well with graduate student milestones (Maughan et al., 2022). Ings observed that faculty can feel apprehensive to provide constructive feedback to students since mesearch topics are so closely tied with personal identities (Ings, 2015). Kolysh (2017) offers a different perspective: “I no longer worry about my methods not being ‘objective’ or that I’m conducting a lot of ‘mesearch.’ That I’m a raging lesbian scholar with collapsed boundaries between my life, teaching, and academics is why I do good work—no one straight or cisgender can get at the intricacies of our LGBTQ communities with as much deference or as much desire for wanting to preserve gender and sexual difference” (Nowakowski and Sumerau, 2017).

While conducting mesearch can be a strong motivator for students to persist in their graduate program, it can also take a mental toll (Hinchey and Kimmel, 2000; Nowakowski and Sumerau, 2017). Koch et al. (2022) described feelings of freedom in combining personal and professional interests (Koch et al., 2022), but Juhasz and Ma (2009) question to what extent these areas should become entwined (Juhasz and Ma, 2009). Students can be professionally pigeonholed (Juhasz and Ma, 2009), and published research on SOGI topics can make both the researcher and their subjects more vulnerable (Ings, 2015).

#### 3.2.8 Queerness vs. professionalism

By engaging in research and teaching, students start developing as academic professionals. As described in Samek and Donofrio, graduate school is a formative period in which students are socialized to the norms of academia (Samek and Donofrio, 2013). In 14 of the studies in this review, LGBTQ+ graduate students experienced conflicts with academia’s expectations of what constitutes professional behavior. Multiple texts described, for example, the way that SGM students were told that their attire or gender presentation were considered unprofessional (Bailey and Miller, 2015; Atherton et al., 2016; Beemyn, 2019; Sokolowski, 2020). Strouse summarizes the central conflict as: “queer identity depends inherently upon resisting normalcy, and therefore, norms are oppressive to queers” (Strouse, 2015). The author further describes ways that graduate programs focus on research methods over fostering curiosity, and a sense of seriousness stifles flamboyance, camp, and irony. Compounding with the broader culture outside of academia, “professional” becomes conflated with “straight” (Cross et al., 2022).

LGBTQ+ students across these studies found that their personal goals came into conflict with the expected goals of graduate education (namely, a focus on research projects and publications), which can feel unfulfilling (Glover, 2017; Duran et al., 2022). These students can enter doctoral programs with different previous professional experiences than their peers (Smith, 1995). Some SGM students valued incorporating activism, DEI work, and community outreach into their graduate school experience, but this was not always appreciated by their program (Brauer et al., 2022; Cisneros et al., 2022; Cross et al., 2022).

#### 3.2.9 Discrimination, harassment, and microaggressions

Beyond exclusionary cultures, SGM doctoral students endured a wide range of anti-LGBTQ+ behaviors in graduate school, and this was one of the most prevalent themes in 46 of the 82 publications. Three publications specifically mentioned physical assaults and threats of violence (Hinchey and Kimmel, 2000; Bailey and Miller, 2015; Gilliam and Swanson, 2020). Overall the authors and participants described their experiences of homophobia (Smith, 1995; Resides, 1997; Hinchey and Kimmel, 2000; Pierce, 2003; Cortez, 2013; Bailey and Miller, 2015; Glover, 2017; Al-Saleh and Noterman, 2021; El Kurd and Hummel, 2023; Reggiani et al., 2023), transphobia (Beemyn, 2019; Gilliam and Swanson, 2020; Maughan et al., 2022), sexism (Cortez, 2013; Al-Saleh and Noterman, 2021; Reggiani et al., 2023), heteronormativity (Freeman, 2018; Ortis, 2018; Singh and Mathews, 2019; Sokolowski, 2020; Al-Saleh and Noterman, 2021), and cisnormativity (Beemyn, 2019; Singh and Mathews, 2019). In one study, 21% of respondents of all SOGIs reported hearing about homophobic incidents, and 13% of LGBTQ+ students reported experiencing homophobia themselves (El Kurd and Hummel, 2023). In another survey of trans and gender nonconforming graduate students, 21% of respondents said their classes were somewhat or very transphobic (Beemyn, 2019).

Many different harassment experiences were described, including sexual harassment by peers (Day, 2010; Atherton et al., 2016), faculty (Nowakowski and Sumerau, 2017), and others (Ortis, 2018; Singh and Mathews, 2019; El Kurd and Hummel, 2023). El Kurd and Hummel found that the trans respondents in their study reported high levels of harassment of multiple types, and they had lower expectations that their department would address reported sexual harassment incidents compared to cis men and cis women (El Kurd and Hummel, 2023). To that point, two of the publications included situations in which a student reported harassment to the university but felt unheard and unsupported afterwards (Day, 2010; Gilliam and Swanson, 2020).

Trans and nonbinary graduate students were persistently misgendered by those around them, including misusing pronouns or refusing to use any pronouns (Beemyn, 2019; Goldberg et al., 2019; Sokolowski, 2020; Knutson et al., 2022; Reggiani et al., 2023). One study reported that 45% of trans and gender nonconforming survey respondents were misgendered by peers often (Beemyn, 2019). Another study looked more closely at students’ academic fields and found that 46% of trans students in the natural sciences reported chronic misgendering compared to 15% in the social sciences (Whitley et al., 2022).

LGBTQ+ students were bullied and defamed by faculty and other students (Misawa, 2009; Cortez, 2013; Gilliam and Swanson, 2020; Doxbeck and Karalis Noel, 2023). For example, Misawa describes an incident with a student affairs staff member at his university: he was seeking help recruiting gay male students of color, and the staff member not only rejected him, but also emailed a campus listserv for students of color and instructed them not to respond to him (Misawa, 2009). Trans people were harassed in bathrooms, including having their picture taken as “evidence” (Beemyn, 2019). Gay couples were intimidated after showing affection to each other at college bars (Cross et al., 2022). LGBTQ+ students were sexualized (Samek and Donofrio, 2013; Ortis, 2018; English and Fenby-Hulse, 2019; Sokolowski, 2020), catcalled (Nowakowski and Sumerau, 2017), victim-blamed (Ortis, 2018), and outed to others (Knutson et al., 2022).

The students in these articles mentioned examples of LGBTQ+ people having unequal access to opportunities or receiving different treatment. Out students received comments about their SOGIs when applying to academic jobs and felt that being out was limiting their opportunities (Smith, 1995; Cisneros et al., 2022). Hinchey and Kimmel (2000) describe a situation in which a student group focused on gay, lesbian, and bisexual issues was redesigned by the faculty administration as a broader diversity group (Hinchey and Kimmel, 2000). As for resources, it was noted that SGM students have inadequate access to physical health care (Knutson et al., 2022).

There were many different experiences described across the texts in which LGBTQ+ graduate students felt excluded or invalidated, sometimes even on a daily basis (Cortez, 2013). Primary among these were instances when people assumed that students were heterosexual (Handy, 2016; Mehra, 2016; Freeman, 2018; Bhattar, 2019; English and Fenby-Hulse, 2019; Duran et al., 2022; Reggiani et al., 2023) or assumed their gender identity from physical appearance (English and Fenby-Hulse, 2019).

Gender dynamics were mentioned often as well, at times amplifying or intersecting with the reported negative experiences of heterosexual and/or cisgender women. Students encountered gendered or exclusionary language regularly (Strouse, 2015), such as “ladies and gentlemen” (Beemyn, 2019) and being described as a “lab mom” (Sokolowski, 2020). In graduate coursework, the content could be presented in a way that reinforced gender binaries, particularly in subjects related to anatomy and languages with gendered words (Goldberg et al., 2021). Professors told students that their preferred pronouns were grammatically incorrect (Goldberg et al., 2021). In two studies, nonbinary and cisgender women students reported that their classrooms tended to be dominated by men who spoke disproportionately frequently and interrupted others (Bailey and Miller, 2015; Strings and Nasir, 2022). Furthermore, female-identified students were told that they should not use research equipment that requires physical strength (Atherton et al., 2016). Multiple trans men shared that cisgender, heterosexual men felt comfortable making disrespectful remarks about women in their presence (Cortez, 2013; Sokolowski, 2020), or otherwise reinforcing problematic masculine stereotypes (Ortis, 2018). When students attempted to discuss the pervasive masculinity in engineering with their cisgender male peers, they were met with derision (Bakka et al., 2021).

The microaggressions in these articles took on many additional forms. SGM students were tokenized (Turkowitz, 2012; Means et al., 2017; Knutson et al., 2022), endured gossip (Ortis, 2018), were held to higher standards or policed for their affect (Glover, 2017), were excluded from conversations and project groups (English and Fenby-Hulse, 2019, Sokolowski, 2020), and were subjected to intrusive questions (Cortez, 2013). The people around them made assumptions that having an LGBTQ+ identity meant having particular political opinions, hobbies, or research interests (Ings, 2015; English and Fenby-Hulse, 2019; Wright-Mair and Marine, 2021). They were told they were the “diversity hire” when applying for academic jobs (Smith, 2014). Mehra (2016) mentions a student whose faculty mentor would pressure trainees of different genders to be romantic partners (Mehra, 2016). On the social side of graduate school, LGBTQ+ students lamented always socializing at bars perceived to be straight spaces (Resides, 1997); conversely, they could also feel pressured by their cisgender-heterosexual peers to go to a drag bar (Sokolowski, 2020). Even with non-SGM peers who wanted to be supportive, LGBTQ+ students endured their peers expressing feelings of straight guilt (Samek and Donofrio, 2013).

Boyle et al. (2022), in particular, go into more detail about the microaggressions and supports experienced by students of intersectional racial and sexual orientation identities (Boyle et al., 2022). One counterpoint was another survey study that found sexual minorities did not report unfair treatment at significantly different rates compared to heterosexual students (Bahnson et al., 2021).

#### 3.2.10 Academic systems and cultures

More broadly, the systems, policies, and cultures of academia affected SGM students’ experiences in graduate school, as was mentioned in 40 of the publications. On the positive side, in English and Fenby-Hulse’s (2019) survey of LGBTQ+ doctoral researchers in the United Kingdom, 72% of respondents agreed or strongly agreed that their department offered an inclusive environment for LGBTQ+ researchers (English and Fenby-Hulse, 2019). However, participants in Resides (1997) study discussed how departments and institutions can have different cultures and how these cultures changed over time (Resides, 1997). For example, during the COVID-19 pandemic, sexual minority doctoral students reported higher levels of mental health symptoms compared to heterosexual respondents, and women and nonbinary doctoral students reported higher workloads compared to men (Douglas et al., 2022; Schad et al., 2022). Institutions are also situated within the regional and national politics of gender, sexual orientation, and other identities (Smith, 1995; Glover, 2017; Sokolowski, 2020).

At the policy level, students experienced both support and additional burdens at their institutions. Some graduate students mentioned receiving help from their programs with changing their name in official records and connecting with campus resources around identity formation (Goldberg et al., 2022). Use of gender-neutral language by universities and colleges was seen as validating (Koch et al., 2022). Other institutional policies created environments in which SGM students’ needs were tolerated, even if they did not feel fully embraced (Resides, 1997; Ings, 2015). Students experienced issues when policies were inflexible to their needs (Cortez, 2013), such as difficulties with health insurance during a medical transition that disrupted academic progress (Heffernan and Gutierez-Schmich, 2016). Institutions may not have procedures for students to share gender identity and pronoun information with faculty and staff (Linley and Kilgo, 2018). In conducting research, students perceived the institution’s priorities from the demographics forms used which may still ask participants’ sex with only binary male/female options (Knutson et al., 2022). More broadly, faculty and staff can be uninformed or silent around these structural inequalities (Heffernan and Gutierez-Schmich, 2016), potentially stemming from a lack of institution-wide initiatives to promote awareness (Reggiani et al., 2023).

While SOGI may have been included in campus diversity statements and protected classes, that did not always translate to adequately inclusive practices (Smith, 1995). Institutions could emphasize diversity broadly, but not commit to supporting LGBTQ+ students more specifically and creating change (Hinchey and Kimmel, 2000; Glover, 2017; Freeman, 2018; Duran et al., 2022). Conversely, there were spaces in which SOGI were treated as a key part of diversity efforts, and SGM students were intentionally included in professional development opportunities (Resides, 1997).

A deep-seated issue that can prevent progress is the emphasis that research needs to be objective and, therefore, distanced from social topics (Strouse, 2015; Cisneros et al., 2022; Cross et al., 2022; Reggiani et al., 2023). Cultures can develop in academic spaces that consider talking about people’s personal lives as a waste of time, uncomfortable, too political, or potentially scandalous (Nowakowski and Sumerau, 2017; Ortis, 2018; Cross et al., 2022). In their coursework, graduate students saw exclusionary language or lack of diversity statements as signals to not speak up (Knutson et al., 2022). Even when LGBTQ+ topics were discussed in class, some students’ non-SGM peers remained disengaged (Samek and Donofrio, 2013). When students explored different fields of study, they could be drawn toward disciplines that felt more open to their identities (Lee, 2017).

LGBTQ+ graduate students observed that stereotypically masculine behaviors were more valued in their departments (Singh and Mathews, 2019; Cross et al., 2022), and not only because some fields have historically been populated by cisgender men (Pierce, 2003). Academic spaces can be high-pressure environments (Pierce, 2003; Doxbeck and Karalis Noel, 2023) with an overt “bro culture” (Reggiani et al., 2023) and competition between researchers (Resides, 1997; Juhasz and Ma, 2009; Smith, 2014; Singh and Mathews, 2019; Wright-Mair and Marine, 2021). Singh and Mathews further connected positive mentoring relationships with stereotypically feminine behaviors: “reciprocity and mutuality of power are central to a practice of feminist co-mentoring, in contrast with the unidirectional top-down configuration of power that often characterizes traditional mentor-mentee relationships” (Singh and Mathews, 2019). There was one counter-narrative in Grunert and Bodner’s (2011) study of women in chemistry: the one lesbian participant had adopted a more traditionally masculine gender role in her romantic relationship, and she did not experience the same structural and cultural impediments to her career as the straight participants (Grunert and Bodner, 2011).

#### 3.2.11 Advocacy: successes and setbacks

To combat the experiences of discrimination, restrictive policies, and unsupportive cultures, SGM graduate students in 23 publications mentioned advocating for themselves and others. They voiced a need for trainings (Beemyn, 2019; Cisneros et al., 2022), inclusive curricula, and equitable resources (Cortez, 2013). They urged their institution to adequately include LGBTQ+ identities as part of campus diversity (Cross et al., 2022). Sometimes advocacy was resisting the established culture of their field (Bakka et al., 2021). Students described impediments they encountered: power structures (Misawa, 2009; Strouse, 2015; Goldberg et al., 2019), research relationships between faculty (Mehra, 2016), and campus offices that tended to exclude graduate students or trans/nonbinary students (Beemyn, 2019).

Most instances of graduate student advocacy in these studies were met with various degrees of resistance. Many experienced retaliation (Mehra, 2016; Glover, 2017; Singh and Mathews, 2019; Cisneros et al., 2022; Knutson et al., 2022), including loss of health insurance and tuition reimbursement (Duran et al., 2022), fewer professional opportunities (Nowakowski and Sumerau, 2017), and exclusion from mentors’ networks (Singh and Mathews, 2019). Others were met with defensiveness, gaslighting, confusion, and invalidation (Smith, 1995; Mehra, 2016; Beemyn, 2019; Gilliam and Swanson, 2020; Sokolowski, 2020; Cross et al., 2022). Advocates could be labeled as troublemakers or killjoys (Glover, 2017; Al-Saleh and Noterman, 2021; Cross et al., 2022). One graduate student mentioned receiving death threats (Cross et al., 2022). Even in programs that seemed receptive to students’ requests, administrators could choose not to enact change (Cortez, 2013; Mehra, 2016). Glover summarizes these situations: “so we keep fighting, knowing that we have no choice, that we will be a perpetual problem, that we may be alone taking these risks” (Glover, 2017).

Some publications included examples in which LGBTQ+ graduate students’ efforts yielded positive results. In Cross et al. (2022), a student convinced friends to stop using the word “gay” as a synonym for “stupid” or “frustrating” (Cross et al., 2022). On a larger scale, Linley and Kilgo (2018) describe a student who received the backing of university leadership to lead a cross-campus effort to improve the university’s record system to be more inclusive of LGBTQ+ identities (Linley and Kilgo, 2018).

#### 3.2.12 Belonging and mental health

The LGBTQ+ graduate students in 38 of these 82 publications made connections among the challenges they faced, feelings of belonging, and their mental health. In surveys, LGBTQ+ graduate students reported lower sense of belonging compared to their non-SGM peers, and they were nearly twice as likely to consider leaving their academic program, a pattern not seen among the undergraduates in the same studies (Stout and Wright, 2016; Platt et al., 2022). Students’ sense of belonging was impacted by experiences of homophobia (Sokolowski, 2020), the emotional labor of coming out repeatedly (Reggiani et al., 2023), dealing with additional burdens within academic systems (Day, 2010; Atherton et al., 2016), comparing themselves with others (Smith, 2014; Cisneros et al., 2022), and feeling uncertain about their own abilities (Pierce, 2003; Smith, 2014). Martinez described the difficulties of becoming the worst-performing student after previously being the best before graduate school (Martinez, 2023). For students engaged in mesearch, their personal connection to the research projects could produce unexpected challenges to their mental health (Handy, 2016). If students experienced tensions between multiple minoritized identities, that could also produce strong imposter feelings (Glover, 2017; Means et al., 2017; Coloma, 2020; Coda, 2023). Juhasz and Ma (2009) even draw parallels between the queer experience in general and imposter feelings (Juhasz and Ma, 2009). Over time, these graduate students can develop a sense of “never being inside,” being repeatedly reminded that they do not belong in academia (Misawa, 2009; Mehra, 2016).

Two studies found that LGBTQ+ doctoral students were more likely to experience mental health conditions compared to their non-SGM peers, such as being six times more likely to report suicidal ideation (Jones-White et al., 2022; Schad et al., 2022). Students in other studies described experiences of suicidal ideation (Hinchey and Kimmel, 2000; Gilliam and Swanson, 2020) and long-lasting trauma after graduate school (Nowakowski and Sumerau, 2017; Ortis, 2018). Some students also saw that their substance use increased during their doctoral programs (Hinchey and Kimmel, 2000; Doxbeck and Karalis Noel, 2023; Gattamorta et al., 2023).

SGM graduate students found ways to combat their imposter feelings and improve their mental health. One approach was to rely on other identity privileges, performing heterosexuality (pretending to be straight), or passing to not draw unwanted attention to themselves (Cross et al., 2022). In the other direction, some graduate students instead embraced authenticity with their identities (Hector et al., 2023). Students found it helpful to read counter-narratives in the literature about other LGBTQ+ people’s experiences (Means et al., 2017). While sharing their personal stories with others can be healing, students described ways in which their stories were consumed and co-opted by their non-SGM peers (Kawano, 2020). Students could reframe their imposter feelings as being an “outsider within” with specific expertise to contribute and agency to foster change (Duran et al., 2022). Glover (2017) highlighted the importance of these steps and the stakes involved: “needing to cultivate self-care, self-love, and differential consciousness to survive, let alone succeed” (Glover, 2017). Engaging in service to the department or community, working with a therapist, and a supportive doctoral advisor were also described as beneficial for mental health (Resides, 1997; Nowakowski and Sumerau, 2017; Cross et al., 2022).

#### 3.2.13 Sources of support

In that vein, students in 47 of the 82 publications mentioned the support they received from individuals or groups during graduate school. In terms of faculty mentors, a survey of doctoral researchers in the United Kingdom found that 75% of respondents felt that their primary supervisor was LGBTQ+ friendly (English and Fenby-Hulse, 2019). A comparable study in the United States observed that while bisexual, lesbian, and gay graduate students reported similar mentoring relationships with faculty advisors compared to heterosexual students, respondents who selected “other” for sexual orientation reported poorer relationships with their advisors (Platt et al., 2022).

The graduate students in these publications found it helpful when their faculty mentors mutually acknowledged their intersectional identities and where there might be limitations in their mentoring relationship (Smith, 1995; Cross et al., 2022). When LGBTQ+ students reveal their identities, supportive faculty reacted with quick acceptance and positivity (Sokolowski, 2020; Koch et al., 2022), followed by an invitation to talk about students’ specific needs (Beemyn, 2019; Cross et al., 2022). In addition to listening to their mentees, students appreciated when faculty sought additional information and resources related to LGBTQ+ experiences (Hector et al., 2023). Discussing strengths rather than only focusing on deficits propelled students’ growth (Means et al., 2017). Students felt understood when faculty used gender-neutral and inclusive language (Smith, 1995; Koch et al., 2022). If mentors made a mistake with language or misgendering, students still felt supported if faculty apologized and self-corrected (Beemyn, 2019; Goldberg et al., 2019; Wright-Mair and Marine, 2021; Koch et al., 2022; Hector et al., 2023). SGM students valued their mentors’ efforts to build their network (Sokolowski, 2020, Cross et al., 2022) and promote their inclusion with the research group (Smith, 1995, Atherton et al., 2016, Wright-Mair and Marine, 2021). As students experienced mistreatment or other crises, faculty who took action were seen as strong allies (Atherton et al., 2016; Cross et al., 2022; Maughan et al., 2022).

Beyond their advisors, LGBTQ+ graduate students received support from a variety of others. Students appreciated finding faculty for their dissertation committees who validated their personal and professional pursuits (Cortez, 2013; Samek and Donofrio, 2013; Lee, 2017; Cisneros et al., 2022) and who shared SOGI or other identities (Singh and Mathews, 2019; Coloma, 2020; Sokolowski, 2020; Wright-Mair and Marine, 2021). Sometimes students had to reach beyond disciplinary and departmental lines to find these faculty (Singh and Mathews, 2019; Sokolowski, 2020). Additional professors and academic staff helped students to navigate the systems of their institution (Wright-Mair and Marine, 2021; Goldberg et al., 2022). Moreover, students felt their identities affirmed by their peers, who often served as their primary social support in graduate school (Levounis, 2003; Ortis, 2018; Stockdill, 2018; Ullman et al., 2018; Wright-Mair and Marine, 2021; Cisneros et al., 2022; Coda, 2023).

In addition, students sought support from communities both on and off campus. Within their fields, students found working groups, conference panels, and mentoring networks (Nadal, 2019; Al-Saleh and Noterman, 2021; Duran et al., 2022). A reading group of LGBTQ+ students and faculty was described by one participant as the “first time I was able to talk freely about my gender and sexual orientation identities in a group of engineers” (Bakka et al., 2021). Campus centers, counseling services, and groups for LGBTQ+ students were seen as resources, particularly when they were welcoming of graduate students (Gould, 1999; Cortez, 2013; Bailey and Miller, 2015; Cross et al., 2022; Doxbeck and Karalis Noel, 2023). Students connected with peers in their doctoral programs who shared one or more identities (Lyle et al., 1999; Juhasz and Ma, 2009; Handy, 2016; Means et al., 2017; Crawley, 2021; Duran et al., 2022; Strings and Nasir, 2022). Outside of academia, students could find validation from family (Grunert and Bodner, 2011; Nowakowski and Sumerau, 2017; Goldberg et al., 2022), community groups (Smith, 1995; Resides, 1997; Gould, 1999), romantic partners (Resides, 1997; Gould, 1999; Means et al., 2017; Duran et al., 2022), and other LGBTQ+ friends (Bailey and Miller, 2015; Ortis, 2018; Sokolowski, 2020). While virtual networks have become more prevalent over time, students said that in-person communities were more affirming (Duran et al., 2022; Gattamorta et al., 2023). Notably, if students have multiple identities historically marginalized in academia, they found it important to balance their social supports across communities of those multiple identities (Bhattar, 2019; Coloma, 2020; Cross et al., 2022).

As a result of these supports, LGBTQ+ students were able to persist through their programs and overcome obstacles (Misawa, 2009; Cortez, 2013; Cross et al., 2022; Doxbeck and Karalis Noel, 2023). Efforts of LGBTQ+ peers and allies served as microaffirmations, helping them to resist the dominant cultures and flourish academically (Bakka et al., 2021; Becerra and Cáraves, 2023; Hector et al., 2023).

## 4 Discussion

This article is the first scoping review to examine the current literature on LGBTQ+ doctoral students. A total of 82 publications were found, and 13 themes emerged. Only 26 publications were found that specifically included students in STEM, so broadening the search strategy to other fields successfully created a richer set of narratives and data for analysis. The included themes are intended to be illustrative of the variety of perspectives present in the literature rather than creating a definitive list. While expanding the literature included beyond STEM and peer-reviewed research studies was an important goal for the scoping review, the emphasis is on the qualitative themes and examples. Most of these texts were published in the past 5 years, and the number of publications per year appears to be increasing in this emerging topic. In the US, lesbian and gay people are attaining bachelor’s and advanced degrees at higher rates than heterosexual people (Mittleman, 2022), but they may not receive the same economic benefits (Mize, 2016). To design and evaluate effective resources and interventions, it is imperative that LGBTQ+ experiences are better understood.

During the timeframe studied, LGBTQ+ communities in the United States, Canada, and Europe experienced substantial increases in visibility, acceptance, and legal protections. More adults were claiming an SGM identity during that time (Twenge et al., 2024), and the number of campuses with LGBTQ+ resource centers increased (Consortium of Higher Education LGBT Resource Professionals, 2024). These trends likely explain the wave of LGBTQ+ scholarship we saw in this study. If SGM students saw academia as more accepting and safe, and there were more visibly out faculty, that could inspire a new generation of research (and mesearch). Increased visibility can also create a backlash, though. The texts in this study described many instances of harassment, microaggressions, and violence, none of which appeared to decrease over the time period studied. A recent study likewise found that more than 1 in 3 LGBTQ+ adults in America have experienced discrimination in the past year (Medina and Mahowald, 2023). Like many historically excluded groups, people of LGBTQ+ identities experience vicissitudes in social support, and future research should examine how these dynamics affect the careers of SGM scholars. For example, how do SGM students choose doctoral programs in the current landscape, and how does this compare to studies on college choice (Nguyen et al., 2022)? If students prioritize programs that are more LGBTQ+-friendly, how does it affect their experiences and career outcomes?

Of the publications that specified the number of LGBTQ+ doctoral students included, half represented the perspectives of five or fewer individuals. Only 9 studies included more than 50 LGBTQ+ doctoral students. Two-thirds of the survey-based publications included in this review did not include the full results for LGBTQ+ doctoral students. Often these data were aggregated: grouping graduate and undergraduate students together; or grouping trans, nonbinary, and female students together. Other times, the data were omitted altogether due to lack of statistical power. Future research should consider the dynamics of studying a relatively small and hard-to-reach population, employing mixed methods approaches (Perrenoud et al., 2023) and more effective sampling methods (Hughes et al., 2021; Raifman et al., 2022).

While some LGBTQ+ graduate students in these publications experienced a lower sense of belonging in academia and imposter feelings, others were able to invert that narrative with a more empowered approach – being an “outsider within.” Some SGM students were able to see these situations as opportunities to educate those around them and question the establishment. At the same time, definitions of “professionalism” can be used to disempower minoritized groups, as has been studied in the context of race (Frye et al., 2020; McCluney et al., 2021). Future research should consider how to shift academic cultures and allow graduate students to contribute more of their individual experiences and interests toward doctoral milestones. For instance, student service or advocacy work could count toward program requirements. Comparable calls have already been made to increase flexibility in faculty tenure and promotion decisions (Trejo, 2020).

Many of these studies discussed the ways in which SGM graduate students conduct mesearch. Only 3 of these studies, though, included STEM students (ex. an engineering PhD student who had interests to study LGBTQ+ identities in their field). This imbalance in academic representation may be historical. Since the 1970s, humanities and social science fields have been acknowledging how intersectional social identities are embedded in the research endeavor (Starfield, 2012), so there likely have been more opportunities to engage with mesearch. One could imagine opportunities for mesearch in STEM fields will grow with increased visibility and acceptance of LGBTQ+ identities, particularly in areas of neuroscience, translational biomedical research, and STEM workplaces and classrooms. It is still worth considering that many of the perspectives in this review shared that their mesearch interests were still devalued. Similar findings have emerged for researchers with personal connections on racial health disparities or mental health conditions (Pololi et al., 2013; Devendorf, 2022). Further research could examine to what extent and in which research areas STEM PhD students are pursuing mesearch projects.

Below, we describe practices that SGM doctoral students in these studies described as beneficial for their persistence, feelings of belonging, and development as professionals. Anyone connected to graduate education has a stake in improving academic environments, including campus leaders, program directors, academic staff, faculty advisors, instructors, and graduate students. For institutions in the United States, recent legislation and campus policies may limit implementation of some of these practices. In some states with DEI restrictions, students may have relatively more freedom than faculty or staff, so some of these recommendations could be implemented by partnering with and empowering student groups. We have included many ideas here on different scales, because even a set of small steps can help students feel supported.

### 4.1 Recommendations for practitioners

1. Openly discuss identities in mentoring relationships: Acknowledge personal identities, pronouns, and experiences from the outset, including areas of mentorship a student is seeking that a mentor is less able to fulfill. Seek out foundational information for straight and cisgender allies (examples from the office of Gender and Sexuality Services at the University of Illinois-Springfield, https://www.uis.edu/gsss/lgbtq-resources/lgbtq-guides/ally-guide/dos-and-donts-straight-allies). Apologize for mistakes made around names, pronouns, or identities, such as examples shown in Cooper et al. (2020).
2. Connect with SGM networks: Offer to help mentees find additional, complementary mentors who can support their identities and professional goals. Inform mentees about field- or career-related professional societies with LGBTQ+ interest groups.
3. Create opportunities for LGBTQ+ visibility: Include pronouns on websites and course rosters. Invite, but do not require, students to share their pronouns during course introductions (example scripts available from the American Psychological Association) (Maroney et al., 2019). Maintain a voluntary Out List of LGBTQ+ students, faculty, and staff who can support current and prospective students (Eckstrand et al., 2017; Awe and Ai, 2022). Invite LGBTQ+-identified researchers to present at departmental seminars, such as those listed at 500 Queer Scientists (https://500queerscientists.com/). Honor Pride Month in June, and assemble informal groups to attend local Pride parades.
4. Create opportunities for ally visibility: Display completed LGBTQ+ trainings in offices or on websites, such as Safe Zone (Katz et al., 2016; Farrell et al., 2017). Include links to local, regional, and national LGBTQ+ resources in student handbooks, on program websites, and in course syllabi.
5. Update course content: In courses that discuss sex and gender, consider more specific terminology to teach the content while not reinforcing cisheterosexism (examples at https://www.genderinclusivebiology.com/). Highlight the work of LGBTQ+ researchers in the field.
6. Gather SOGI data: Currently, few institutions and programs consistently collect any SOGI data (Freeman, 2020). Include SOGI questions in forms and applications similar to other demographics. Periodically analyze student (and faculty and employee) outcomes by SOGI groups. In programs with sufficiently large cohorts, share aggregated SOGI data with current and potential students. Additional guidance is available from the National Academies of Sciences, Engineering, and Medicine (2022).
7. Use inclusive research methodologies: Depending on the scope and design of a study, it may be challenging to recruit sufficient LGBTQ+ participants to analyze their data as a group (let alone comparing specific identities within that umbrella). Utilize mixed methods approaches, such as surveys along with interviews/focus groups or even open-ended survey questions so that SGM participants’ perspectives can still be included if sample sizes are small (Perrenoud et al., 2023). Consider different sampling methodologies and partnerships with LGBTQ+ communities (Hughes et al., 2021, Raifman et al., 2022).
8. Train graduate student instructors to handle inappropriate classroom conduct: Provide guidance and practice opportunities for responding to discriminatory behaviors in the classroom, either directed at them or at other students. Identify faculty and academic staff who can support them in these situations.
9. Promote LGBTQ+ groups and organizations: Host student groups and professional organizations in classes. Provide lists of LGBTQ+ groups (such as oSTEM chapters, https://ostem.org/) to all students. Fund groups to host events and travel to conferences. Attend the groups’ events, such as invited seminars, journal clubs, and celebrations.
10. Foster inclusive and safe academic cultures: Examine the ways in which current departmental, campus, and field-specific cultures reinforce cisheterosexism and incentivize behaviors stereotypically associated with masculinity. Ensure reporting systems for violence, harassment, and mistreatment support LGBTQ+ victims. Regularly send relevant resources and information to faculty to use in their teaching and mentoring.

### 4.2 Limitations

Limitations of this review derive primarily from the selection criteria employed. By excluding publications about clinical fields, there may be unique experiences from client-serving disciplines missing from our themes. Selecting texts published in English also removed potential perspectives from communities or countries that may not be represented, though only three publications were removed for this reason during screening.

### 4.3 Conclusion

Overall, this scoping review is intended as a starting point for practitioners who teach and mentor SGM graduate students. More research is urgently needed to better understand and improve these students’ experiences. The themes, methodologies, and ideas presented can propel future endeavors to create impactful practices and resources, thereby making academia a more inclusive environment for individuals who have historically been marginalized.

## Supplementary Material

Supplemental Table 1

Supplemental Data Sheet 1

Supplemental Table 2

The Supplementary material for this article can be found online at: https://www.frontiersin.org/articles/10.3389/feduc.2024.1472113/full#supplementary-material

## Figures and Tables

**TABLE 1:** Emergent themes from included studies in the order they appear in this publication.

Theme	Number of publications containing theme
Identities under formation	28
Intersectional identities	36
Motivations for graduate studies	7
Unmet expectations of graduate school	14
Coming out and safety	45
Teaching and management relationships	13
Mesearch	32
Queerness vs. professionalism	14
Discrimination, harassment, and microaggressions	46
Academic systems and cultures	40
Advocacy	23
Belonging and mental health	38
Sources of support	47

**TABLE 2:** Publications mentioning intersectional experiences, grouped by type of identity.

Identity category	Publications
Race/ethnicity	Resides (1997), Lyle et al. (1999), Cortez (2013), Samek and Donofrio (2013), Smith (2014), Bailey and Miller (2015), Handy (2016), Mehra (2016), Glover (2017), Lee (2017), Means et al. (2017), Ortis (2018), Nadal (2019), Singh and Mathews (2019), Kawano (2020), Wright-Mair and Marine (2021), Boyle et al. (2022), Cisneros et al. (2022), Cross et al. (2022), Duran et al. (2022), Platt et al. (2022), Strings and Nasir (2022), and Becerra and Cáraves (2023)
Socioeconomic status/class	Samek and Donofrio (2013), Smith (2014), Means et al. (2017), Ullman et al. (2018), Coloma (2020), and Coda (2023)
International status	Lyle et al. (1999), Misawa (2009), Bhattar (2019), Coloma (2020), and Martinez (2023)
Disability	Levounis (2003), Samek and Donofrio (2013), Ullman et al. (2018), Whitley et al. (2022), and Reggiani et al. (2023)
Religion	Resides (1997), Barbier (2007), Cortez (2013), and Cross et al. (2022)
US region of origin	Cortez (2013), Glover (2017), and Ullman et al. (2018)
First-generation	Whitley et al. (2022)
Neurodiversity	Duran et al. (2022)
Parental status	Cross et al. (2022)
Polyamorous	Ortis (2018)
Rural area of origin	Duran et al. (2022)
