# Supplementary material for: Experiences of LGBTQ+ graduate students in research-focused doctoral programs: a scoping review
Source: Front Educ (Lausanne). Author manuscript; Available in PMC 2026 Jan 31. (PMC12858023; doi:10.3389/feduc.2024.1472113)
Supplement: Supplemental Table 1 [file NIHMS2125844-supplement-Supplemental_Table_1.docx]

**Supplemental Table 1. Characteristics of Included Studies (N = 82)**

| **Publication** | **Type** | **Methodology** | **Field(s)** | **No. SGM Doctoral Students* / Total No. Participants** | **Theoretical Frameworks** |
| --- | --- | --- | --- | --- | --- |
| Al-Saleh & Noterman, 2021 | Peer-reviewed article | Survey, reflection, intervention | Physical sciences, social sciences | Not provided | Not specified |
| Atherton et al., 2016 | Society report | Survey, focus group, interview | Physical sciences | 126 / 324 | Not specified |
| Bahnson et al., 2021 | Conference paper | Survey | Engineering | 26 / 250 | Not specified |
| Bailey & Miller, 2015 | Peer-reviewed article | Interview, reflection, transcribed conversation | Social sciences | 2 / 2 | Feminist theory and praxis |
| Bakka et al., 2021 | Conference paper | Survey, reflection | Engineering | 3 / 8 | Not specified |
| Barbier, 2007 | Dissertation | Text analysis | Education | 1 / 1 | Identity status paradigm, poststructuralism, identity politics, genre theory |
| Barnes et al., 2021 | Peer-reviewed article | Survey, interview | Life sciences | 8 / 33 | Not specified |
| Becerra & Cáraves, 2023 | Peer-reviewed article | Reflection | Social sciences | 2 / 2 | Jotería studies, critical race theory |
| Beemyn, 2019 | Book | Survey, interview, reflection | Education, humanities and arts, social sciences | Not provided / 93; 1 / 1 (each for 2 chapters)^#^ | Not specified |
| Bhattar, 2019 | Dissertation | Survey, interview, phenomenological research | Social sciences | 1 / 4 | Not specified |
| Boyle et al., 2022 | Peer-reviewed article | Survey | Not specified | Not provided / 2,054 | Minority stress theory |
| Brauer et al., 2022 | Peer-reviewed article | Survey | Physical sciences | 33 / 180 | Cultural capital, social reproduction, critical race theory |
| Burford, 2017 | Peer-reviewed article | Interview, reflection | Education, humanities and arts | 1 / 10 | Queer theory |
| Cisneros et al., 2022 | Book | Autoethnography, duoethnography, reflection | Social sciences, humanities and arts, education | 2 / 2; 1 / 2 (each for 2 chapters); 3 / 3; not provided in some chapters^#^ | Not specified |
| Coda, 2023 | Book chapter | Autoethnography, reflection | Education, humanities and arts | 1 / 1 | Poststructuralism, queer theory |
| Coloma, 2020 | Peer-reviewed article | Reflection | Humanities and arts | 1 / 1 | Women of color feminism |
| Cortez, 2013 | Dissertation | Interview | Social sciences, humanities and arts | 2 / 8 | Critical narrative research, decolonizing methodologies |
| Crawley, 2021 | Book chapter | Reflection | Education | 1 / 1 | Queer theory, hope theory |
| Cross et al., 2022 | Book | Autoethnography, reflection | Engineering | 1 / 1 each for 7 chapters^#^ | Not specified |
| Day, 2010 | Peer-reviewed article | Autoethnography, performative writing | Humanities and arts | 1 / 1 | Not specified |
| Douglas et al., 2022 | Peer-reviewed article | Survey | Life sciences, physical sciences, social sciences | Not provided / 3,243 | Not specified |
| Doxbeck & Karalis Noel, 2023 | Peer-reviewed article | Survey, interview | Engineering, computational fields, social sciences, education, humanities and arts | 2-4 / 10 | Contemporary critical theory |
| Duran, 2021 | Book chapter | Reflection | Education | 1 / 1 | Not specified |
| Duran et al., 2022 | Book | Autoethnography, interview, reflection | Education, social sciences, humanities and arts | 2 / 2 (each for 3 chapters); 1 / 1 (each for 2 chapters)^#^ | Not specified |
| El Kurd & Hummel, 2023 | Peer-reviewed article | Survey | Social sciences | 53-63 / 205 | Not specified |
| English & Fenby-Hulse, 2019 | Peer-reviewed article | Survey | Not specified | 224 / 224 | Not specified |
| Freeman, 2018 | Editorial | Reflection | STEM | 1 / 1 | Not specified |
| Gattamorta et al., 2023 | Peer-reviewed article | Survey | Not specified | 81 / 565 | Not specified |
| Gilliam & Swanson, 2020 | Peer-reviewed article | Reflection | Social sciences | 1 / 2 | Not specified |
| Glover, 2017 | Peer-reviewed article | Reflection | Humanities and arts | 1 / 1 | Black lesbian/queer positionality |
| Goldberg et al., 2019 | Peer-reviewed article | Survey | Life sciences, physical sciences, computational fields, social sciences, humanities and arts, education | 2+ / 91 | Queer, microaggressions, and minority stress frameworks |
| Goldberg et al., 2021 | Peer-reviewed article | Survey, interview | Physical sciences, computational fields, social sciences, humanities and arts, education | Not provided / 30 | Person-environment fit theories of career decision-making, minority stress theory, theory of work adjustment |
| Goldberg et al., 2022 | Peer-reviewed article | Survey, interview | Physical sciences, computational fields, social sciences, humanities and arts, education | Not provided / 30 | Educational decision-making theories, gender minority stress theory, Gati, Krausz, and Osipow's model of career decision-making |
| Gould, 1999 | Book chapter | Case report | Social sciences | 1 / 1 | Not specified |
| Grunert & Bodner, 2011 | Peer-reviewed article | Interview | Physical sciences | 1 / 10 | Standpoint feminism |
| Handy, 2016 | Dissertation | Interview | Education | 1 / 4 | Not specified |
| Hector et al., 2023 | Peer-reviewed article | Reflection | Education | 1 / 3 | Not specified |
| Heffernan & Gutierez-Schmich, 2016 | Book chapter | Survey | Education | 1 / 2 | Not specified |
| Hinchey & Kimmel, 2000 | Book | Interview, reflection | Not specified | Not provided | Not specified |
| Ings, 2015 | Peer-reviewed article | Interview, observations, text analysis | Humanities and arts | Not provided | Not specified |
| Jones-White et al., 2022 | Peer-reviewed article | Survey | Life sciences, physical sciences, engineering, computational fields, social sciences, humanities and arts, education | 290-324 / 2,582 | Occupational health research idea that "work organization and health are highly intertwined" |
| Juhasz & Ma, 2009 | Peer-reviewed article | Reflection | Humanities and arts | 4 / 5 | Not specified |
| Kawano, 2020 | Dissertation | Focus group, interview | Social sciences, humanities and arts | 1 / 9 | Anti-colonial, anti-racist, and feminist |
| Knutson et al., 2022 | Peer-reviewed article | Narrative review | Not specified | Not provided | Socio-ecological model |
| Koch et al., 2022 | Peer-reviewed article | Survey, focus group | Not specified | 1-4 / 13 | Not specified |
| Lee, 2017 | Dissertation | Interview | Social sciences, humanities and arts | 1 / 8 | Not specified |
| Levounis, 2003 | Book chapter | Case report | Humanities and arts | 1 / 1 | Not specified |
| Linley & Kilgo, 2018 | Peer-reviewed article | Reflection | Education | 1 / 2 | Not specified |
| Lyle et al., 1999 | Peer-reviewed article | Interview, reflection | Social sciences | 1 / 3 | Not specified |
| Martinez, 2023 | Peer-reviewed article | Interview, ethnography | Humanities and arts | 2 / 4 | Not specified |
| Maughan et al., 2022 | Peer-reviewed article | Autoethnography | Education | 1 / 1 | Institutional transphobia |
| McNaron, 1996 | Book | Reflection | Not specified | Not provided | Not specified |
| Means et al., 2017 | Peer-reviewed article | Reflection, panel discussion | Social sciences, education | 5 / 5 | Intersectionality, Black queer studies, quare theory |
| Mehra, 2016 | Peer-reviewed article | Reflection | Humanities and arts | 1 / 1 | Not specified |
| Mintz & Rothblum, 1997 | Book | Reflection | Social sciences | 1 / 1 each for 2 chapters^#^ | Not specified |
| Misawa, 2009 | Peer-reviewed article | Reflection | Education | 1 / 1 | Not specified |
| Nadal, 2019 | Peer-reviewed article | Survey | Social sciences | 129 / 368 | Pedagogy of the Oppressed, queer theory, intersectionality |
| Nowakowski & Sumerau, 2017 | Book | Reflection | Social sciences, humanities and arts | 1 / 1 each for 2 chapters^#^ | Not specified |
| Ortis, 2018 | Dissertation | Interview, survey | Not specified | 1+ / 7 | Not specified |
| Phillips, 2018 | Editorial | Reflection | Life sciences | 1 / 1 | Not specified |
| Pierce, 2003 | Peer-reviewed article | Reflection | Social sciences | 1 / 1 | Not specified |
| Platt et al., 2022 | Peer-reviewed article | Survey | Life sciences, physical sciences, social sciences | 240-262 / 1,272 | Not specified |
| Reggiani et al., 2023 | Peer-reviewed article | Focus group, interview, autoethnography | Life sciences, physical sciences, engineering, social sciences | 16-19 / 24 | Intersectionality, post-structural, queer theory |
| Resides, 1997 | Dissertation | Interview | Social sciences, education | 7 / 7 | Postmodern feminist theory |
| Rushworth et al., 2021 | Peer-reviewed article | Survey | Life sciences | 108-121 / 852 | Not specified |
| Samek & Donofrio, 2013 | Peer-reviewed article | Reflection | Humanities and arts | 1 / 2 | Queer theory |
| Satterfield et al., 2019 | Conference paper | Survey | Engineering | 18 / 1,482 | Identity-based motivation |
| Schad et al., 2022 | Peer-reviewed article | Survey | Life sciences | 60+ / 957 | Not specified |
| Singh & Mathews, 2019 | Peer-reviewed article | Autoethnography, reflection | Social sciences | 2 / 2 | Not specified |
| Sloane, 1993 | Book chapter | Interview, reflection | Humanities & arts | 1 / 1 | Not specified |
| Smith, 1995 | Peer-reviewed article | Narrative review | Not specified | Not provided | Not specified |
| Smith, 2014 | Book chapter | Reflection | Social sciences | 1 / 1 | Not specified |
| Sokolowski, 2020 | Dissertation | Interview | Physical sciences | 3 / 3 | Queer theory |
| Stockdill, 2018 | Peer-reviewed article | Reflection, lecture notes | Social sciences | 1 / 1 | Queer theory, black feminism |
| Stout & Wright, 2016 | Peer-reviewed article | Survey | Computational fields | Not specified / 994 | Not specified |
| Strings & Nasir, 2022 | Peer-reviewed article | Survey | Social sciences | 3 / 22 | Not specified |
| Strouse, 2015 | Peer-reviewed article | Reflection | Humanities & arts | 1 / 1 | Not specified |
| Turkowitz, 2012 | Dissertation | Survey, focus group, interview | Life sciences, physical sciences, computational fields, humanities and arts | 7 / 17 | Not specified |
| Ullman et al., 2018 | Book chapter | Reflection | Education | 1 / 1 | Not specified |
| Whitley et al., 2022 | Peer-reviewed article | Survey | Life sciences, physical sciences, social sciences | Not provided / 245 | Not specified |
| Wright-Mair & Marine, 2021 | Peer-reviewed article | Interview | Not specified | Not provided / 11 | Intersectionality theory |
| Yitmen & Almusaed, 2022 | Conference paper | Narrative review | Social sciences | Not provided | Not specified |

* fitting inclusion criteria for this review. ^#^ numbers for books reported for each relevant chapter.
