# Supplementary material for: Experiences of LGBTQ+ graduate students in research-focused doctoral programs: a scoping review
Source: Front Educ (Lausanne). Author manuscript; Available in PMC 2026 Jan 31. (PMC12858023; doi:10.3389/feduc.2024.1472113)
Supplement: Supplemental Data Sheet 1 [file NIHMS2125844-supplement-Supplemental_Data_Sheet_1.docx]

# Supplemental Figure 1. Detailed Search Strategy.

## Databases

| **Database** | **Number of results** | **Unique results** |
| --- | --- | --- |
| PubMed (NLM) | 487 | 487 |
| Scopus (Elsevier) | 659 | 502 |
| ERIC (EBSCOhost) | 323 | 259 |
| PsycInfo (EBSCOhost) | 712 | 478 |
| Academic Search Premier (EBSCOhost) | 437 | 232 |
| Citation searching | 16 | 13 |
|  | 2,634 | 1,971 |

## PubMed (National Library of Medicine)

Search conducted 6/28/23

|  | Search String | Number of Results |
| --- | --- | --- |
| 1 | 2SLGBTQIA[tw] OR agender[tw] OR asexual[tw] OR asexuality[tw] OR asexuals[tw] OR biphobia[tw] OR bisexual[tw] OR "bisexuality"[mesh] OR bisexuality[tw] OR bisexuals[tw] OR cissexism[tw] OR cissexist[tw] OR gay[tw] OR gayness[tw] OR gays[tw] OR "gender diverse"[tw] OR "gender diversity"[tw] OR "gender dysphoria"[tw] OR "gender expression*"[tw] OR "gender identity"[tw] OR "gender minorities"[tw] OR "gender minority"[tw] OR "gender nonconforming"[tw] OR "gender non-conforming"[tw] OR "gender queer"[tw] OR "gender transition"[tw] OR "gender variant"[tw] OR genderqueer[tw] OR GLBT[tw] OR GLBTQ[tw] OR heteronormative[tw] OR heteronormativity[tw] OR homophobia[tw] OR homophobic[tw] OR homosexual[tw] OR "homosexuality"[mesh] OR homosexuality[tw] OR "homosexuality, female"[mesh] OR "homosexuality, male"[mesh] OR homosexuals[tw] OR intersex[tw] OR lesbian[tw] OR lesbianism[tw] OR lesbians[tw] OR LGBTQ[tw] OR LGBTQIA[tw] OR "men having sex with men"[tw] OR "men who have sex with men"[tw] OR misgender[tw] OR misgendered[tw] OR misgendering[tw] OR nonbinary[tw] OR non-binary[tw] OR pansexual[tw] OR pansexuality[tw] OR queer[tw] OR queerness[tw] OR queers[tw] OR same gender couple[tw] OR "same gender couples"[tw] OR "same gender loving"[tw] OR "same sex attracted"[tw] OR "same sex attraction"[tw] OR "same sex couple"[tw] OR "same sex couples"[tw] OR "sexual and gender minorities"[mesh] OR "sexual and gender minority"[tw] OR "sexual identity"[tw] OR "sexual minorities"[tw] OR "sexual minority"[tw] OR "sexual orientation"[tw] OR "sexual preference"[tw] OR TGNC[tw] OR "third sex"[tw] OR "trans female"[tw] OR "trans male"[tw] OR "trans man"[tw] OR "trans men"[tw] OR "trans people"[tw] OR "trans person"[tw] OR "trans woman"[tw] OR "trans women"[tw] OR transexuality[tw] OR transexuals[tw] OR transgender[tw] OR "transgender persons"[mesh] OR transgendered[tw] OR transness[tw] OR transphobia[tw] OR transphobic[tw] OR transsexual[tw] OR "transsexualism"[mesh] OR "two-spirit"[tw] OR "women having sex with women"[tiab:~0] OR "women loving women"[tw] OR "women who have sex with women"[tw] | 101,608 |
| 2 | "doctor of philosophy degree"[tw] OR "doctoral degree"[tw] OR "doctoral degrees"[tw] OR "doctoral education"[tw] OR "doctoral program"[tw] OR "doctoral programs"[tw] OR "doctoral student"[tw] OR "doctoral students"[tw] OR "education, graduate"[mesh] OR "graduate education"[tw] OR "graduate program"[tw] OR "graduate programs"[tw] OR "graduate school"[tw] OR "graduate schools"[tw] OR "graduate student"[tw] OR "graduate students"[tw] OR "graduate study"[tw] OR "graduate training"[tw] OR "PhD education"[tw] OR "PhD program"[tw] OR "PhD programs"[tw] OR "PhD student"[tw] OR "PhD students"[tw] OR "PhD training"[tw] OR "postbaccalaureate"[tw] OR "Postgraduate students"[tw] | 107,983 |
| 3 | #1 AND #2 | **487** |

## SCOPUS (Elsevier)

Search conducted 6/28/23

|  | **Search string** | **Number of results** |
| --- | --- | --- |
| 1 | TITLE-ABS-KEY(2SLGBTQIA OR agender OR asexual OR asexuality OR asexuals OR biphobia OR bisexual OR bisexuality OR bisexuals OR cissexism OR cissexist OR gay OR gayness OR gays OR "gender diverse" OR "gender diversity" OR "gender dysphoria" OR "gender expression*" OR "gender identity" OR "gender minorities" OR "gender minority" OR "gender nonconforming" OR "gender non-conforming" OR "gender queer" OR genderqueer OR "gender variant" OR GLBT OR GLBTQ OR heteronormative OR heteronormativity OR homophobia OR homophobic OR homosexual OR homosexuality OR homosexuals OR intersex OR lesbian OR lesbianism OR lesbians OR LGBTQ OR LGBTQIA OR "men having sex with men" OR "men who have sex with men" OR misgender OR misgendered OR misgendering OR nonbinary OR non-binary OR pansexual OR pansexuality OR queer OR queerness OR queers OR "same gender couple" OR "same gender couples" OR "same gender loving" OR "same sex attracted" OR "same sex attraction" OR "same sex couple" OR "same sex couples" OR "sexual and gender minorities" OR "sexual and gender minority" OR "sexual identity" OR "sexual minorities" OR "sexual minority" OR "sexual orientation" OR "sexual preference" OR TGNC OR "third sex" OR "trans female" OR "trans male" OR "trans man" OR "trans men" OR "trans people" OR "trans person" OR "trans woman" OR "trans women" OR transexuality OR transexuals OR transgender OR transgendered OR transness OR transphobia OR transphobic OR transsexual OR transsexualism OR two-spirit OR "women having sex with women" OR "women loving women" OR "women who have sex with women") | 205,435 |
| 2 | TITLE-ABS-KEY("doctor of philosophy degree" OR "doctoral degree" OR "doctoral degrees" OR "doctoral education" OR "doctoral program" OR "doctoral programs" OR "doctoral student" OR "doctoral students" OR "graduate education" OR "graduate program" OR "graduate programs" OR "graduate school" OR "graduate schools" OR "graduate student" OR "graduate students" OR "graduate study" OR "graduate training" OR "PhD education" OR "PhD program" OR "PhD programs" OR "PhD student" OR "PhD students" OR "PhD training" OR postbaccalaureate OR "Postgraduate students") | 83,450 |
| 3 | 1 AND 2 | 659 |
|  |  |  |

## ERIC (EBSCOhost)

Search conducted 6/28/23

| **Order** | **Search string** | **Number of results** |
| --- | --- | --- |
| 1 | DE "homosexuality" OR DE "LGBTQ People" OR DE "sexual identity" OR DE “sexual orientation" OR (TI(2SLGBTQIA OR agender OR asexual OR asexuality OR asexuals OR biphobia OR bisexual OR bisexuality OR bisexuals OR cissexism OR cissexist OR gay OR gayness OR gays OR "gender diverse" OR "gender diversity" OR "gender dysphoria" OR "gender expression*" OR "gender identity" OR "gender minorities" OR "gender minority" OR "gender nonconforming" OR "gender non-conforming" OR "gender queer" OR genderqueer OR "gender transition" OR "gender variant" OR GLBT OR GLBTQ OR heteronormative OR heteronormativity OR homophobia OR homophobic OR homosexual OR homosexuality OR homosexuals OR intersex OR lesbian OR lesbianism OR lesbians OR LGBTQ OR LGBTQIA OR "men having sex with men" OR "men who have sex with men" OR misgender OR misgendered OR misgendering OR nonbinary OR non-binary OR pansexual OR pansexuality OR queer OR queerness OR queers OR "same gender couple" OR "same gender couples" OR "same gender loving" OR "same sex attracted" OR "same sex attraction" OR "same sex couple" OR "same sex couples" OR "sexual and gender minorities" OR "sexual and gender minority" OR "sexual identity" OR "sexual minorities" OR "sexual minority" OR "sexual orientation" OR "sexual preference" OR TGNC OR "third sex" OR "trans female" OR "trans male" OR "trans man" OR "trans men" OR "trans people" OR "trans person" OR "trans woman" OR "trans women" OR transexuality OR transexuals OR transgender OR transgendered OR transness OR transphobia OR transphobic OR transsexual OR transsexualism OR two-spirit OR "women having sex with women" OR "women loving women" OR "women who have sex with women")) OR (AB(2SLGBTQIA OR agender OR asexual OR asexuality OR asexuals OR biphobia OR bisexual OR bisexuality OR bisexuals OR cissexism OR cissexist OR gay OR gayness OR gays OR "gender diverse" OR "gender diversity" OR "gender dysphoria" OR "gender expression*" OR "gender identity" OR "gender minorities" OR "gender minority" OR "gender nonconforming" OR "gender non-conforming" OR "gender queer" OR genderqueer OR "gender transition" OR "gender variant" OR GLBT OR GLBTQ OR heteronormative OR heteronormativity OR homophobia OR homophobic OR homosexual OR homosexuality OR homosexuals OR intersex OR lesbian OR lesbianism OR lesbians OR LGBTQ OR LGBTQIA OR "men having sex with men" OR "men who have sex with men" OR misgender OR misgendered OR misgendering OR nonbinary OR non-binary OR pansexual OR pansexuality OR queer OR queerness OR queers OR "same gender couple" OR "same gender couples" OR "same gender loving" OR "same sex attracted" OR "same sex attraction" OR "same sex couple" OR "same sex couples" OR "sexual and gender minorities" OR "sexual and gender minority" OR "sexual identity" OR "sexual minorities" OR "sexual minority" OR "sexual orientation" OR "sexual preference" OR TGNC OR "third sex" OR "trans female" OR "trans male" OR "trans man" OR "trans men" OR "trans people" OR "trans person" OR "trans woman" OR "trans women" OR transexuality OR transexuals OR transgender OR transgendered OR transness OR transphobia OR transphobic OR transsexual OR transsexualism OR two-spirit OR "women having sex with women" OR "women loving women" OR "women who have sex with women")) OR (SU(2SLGBTQIA OR agender OR asexual OR asexuality OR asexuals OR biphobia OR bisexual OR bisexuality OR bisexuals OR cissexism OR cissexist OR gay OR gayness OR gays OR "gender diverse" OR "gender diversity" OR "gender dysphoria" OR "gender expression*" OR "gender identity" OR "gender minorities" OR "gender minority" OR "gender nonconforming" OR "gender non-conforming" OR "gender queer" OR genderqueer OR "gender transition" OR "gender variant" OR GLBT OR GLBTQ OR heteronormative OR heteronormativity OR homophobia OR homophobic OR homosexual OR homosexuality OR homosexuals OR intersex OR lesbian OR lesbianism OR lesbians OR LGBTQ OR LGBTQIA OR "men having sex with men" OR "men who have sex with men" OR misgender OR misgendered OR misgendering OR nonbinary OR non-binary OR pansexual OR pansexuality OR queer OR queerness OR queers OR "same gender couple" OR "same gender couples" OR "same gender loving" OR "same sex attracted" OR "same sex attraction" OR "same sex couple" OR "same sex couples" OR "sexual and gender minorities" OR "sexual and gender minority" OR "sexual identity" OR "sexual minorities" OR "sexual minority" OR "sexual orientation" OR "sexual preference" OR TGNC OR "third sex" OR "trans female" OR "trans male" OR "trans man" OR "trans men" OR "trans people" OR "trans person" OR "trans woman" OR "trans women" OR transexuality OR transexuals OR transgender OR transgendered OR transness OR transphobia OR transphobic OR transsexual OR transsexualism OR two-spirit OR "women having sex with women" OR "women loving women" OR "women who have sex with women")) | 10,842 |
| 2 | DE “doctoral degrees” OR DE “doctoral programs” OR DE “doctoral students” OR DE “graduate study” OR DE “Graduate Students” OR (TI("doctor of philosophy degree" OR "doctoral degree" OR "doctoral degrees" OR "doctoral education" OR "doctoral program" OR "doctoral programs" OR "doctoral student" OR "doctoral students" OR "graduate education" OR "graduate program" OR "graduate programs" OR "graduate school" OR "graduate schools" OR "graduate student" OR "graduate students" OR "graduate study" OR "graduate training" OR "PhD education" OR "PhD program" OR "PhD programs" OR "PhD student" OR "PhD students" OR "PhD training" OR postbaccalaureate OR "Postgraduate students")) OR (AB("doctor of philosophy degree" OR "doctoral degree" OR "doctoral degrees" OR "doctoral education" OR "doctoral program" OR "doctoral programs" OR "doctoral student" OR "doctoral students" OR "graduate education" OR "graduate program" OR "graduate programs" OR "graduate school" OR "graduate schools" OR "graduate student" OR "graduate students" OR "graduate study" OR "graduate training" OR "PhD education" OR "PhD program" OR "PhD programs" OR "PhD student" OR "PhD students" OR "PhD training" OR postbaccalaureate OR "Postgraduate students")) OR (SU("doctor of philosophy degree" OR "doctoral degree" OR "doctoral degrees" OR "doctoral education" OR "doctoral program" OR "doctoral programs" OR "doctoral student" OR "doctoral students" OR "graduate education" OR "graduate program" OR "graduate programs" OR "graduate school" OR "graduate schools" OR "graduate student" OR "graduate students" OR "graduate study" OR "graduate training" OR "PhD education" OR "PhD program" OR "PhD programs" OR "PhD student" OR "PhD students" OR "PhD training" OR postbaccalaureate OR "Postgraduate students")) | 50,229 |
| 3 | 1 AND 2 | 323 |

## PsycINFO (EBSCOhost)

Search conducted 6/28/23

| **Order** | **Search string** | **Number of results** |
| --- | --- | --- |
| 1 | DE "asexuality" OR DE "Bisexuality" OR DE "Gender Dysphoria" OR DE "Gender Expression" OR DE "gender identity" OR DE "Gender Nonbinary" OR DE "gender nonconforming" OR DE "Gender Transition" OR DE "homosexuality" OR DE "Homosexuality (Attitudes Toward)" OR DE "intersex" OR DE "lesbianism" OR DE "LGBTQ" OR DE "Male Homosexuality" OR DE "Pansexuality" OR DE "same sex couples" OR DE "sexual identity" OR DE "Sexual Minority Groups" OR DE "Sexual Orientation" OR DE "Transgender" OR DE "Transsexualism" OR DE "Two-Spirit" OR (TI(2SLGBTQIA OR agender OR asexual OR asexuality OR asexuals OR biphobia OR bisexual OR bisexuality OR bisexuals OR cissexism OR cissexist OR gay OR gayness OR gays OR "gender diverse" OR "gender diversity" OR "gender dysphoria" OR "gender expression*" OR "gender identity" OR "gender minorities" OR "gender minority" OR "gender nonconforming" OR "gender non-conforming" OR "gender queer" OR genderqueer OR "gender transition" OR "gender variant" OR GLBT OR GLBTQ OR heteronormative OR heteronormativity OR homophobia OR homophobic OR homosexual OR homosexuality OR homosexuals OR intersex OR lesbian OR lesbianism OR lesbians OR LGBTQ OR LGBTQIA OR "men having sex with men" OR "men who have sex with men" OR misgender OR misgendered OR misgendering OR nonbinary OR non-binary OR pansexual OR pansexuality OR queer OR queerness OR queers OR "same gender couple" OR "same gender couples" OR "same gender loving" OR "same sex attracted" OR "same sex attraction" OR "same sex couple" OR "same sex couples" OR "sexual and gender minorities" OR "sexual and gender minority" OR "sexual identity" OR "sexual minorities" OR "sexual minority" OR "sexual orientation" OR "sexual preference" OR TGNC OR "third sex" OR "trans female" OR "trans male" OR "trans man" OR "trans men" OR "trans people" OR "trans person" OR "trans woman" OR "trans women" OR transexuality OR transexuals OR transgender OR transgendered OR transness OR transphobia OR transphobic OR transsexual OR transsexualism OR two-spirit OR "women having sex with women" OR "women loving women" OR "women who have sex with women")) OR (AB(2SLGBTQIA OR agender OR asexual OR asexuality OR asexuals OR biphobia OR bisexual OR bisexuality OR bisexuals OR cissexism OR cissexist OR gay OR gayness OR gays OR "gender diverse" OR "gender diversity" OR "gender dysphoria" OR "gender expression*" OR "gender identity" OR "gender minorities" OR "gender minority" OR "gender nonconforming" OR "gender non-conforming" OR "gender queer" OR genderqueer OR "gender transition" OR "gender variant" OR GLBT OR GLBTQ OR heteronormative OR heteronormativity OR homophobia OR homophobic OR homosexual OR homosexuality OR homosexuals OR intersex OR lesbian OR lesbianism OR lesbians OR LGBTQ OR LGBTQIA OR "men having sex with men" OR "men who have sex with men" OR misgender OR misgendered OR misgendering OR nonbinary OR non-binary OR pansexual OR pansexuality OR queer OR queerness OR queers OR "same gender couple" OR "same gender couples" OR "same gender loving" OR "same sex attracted" OR "same sex attraction" OR "same sex couple" OR "same sex couples" OR "sexual and gender minorities" OR "sexual and gender minority" OR "sexual identity" OR "sexual minorities" OR "sexual minority" OR "sexual orientation" OR "sexual preference" OR TGNC OR "third sex" OR "trans female" OR "trans male" OR "trans man" OR "trans men" OR "trans people" OR "trans person" OR "trans woman" OR "trans women" OR transexuality OR transexuals OR transgender OR transgendered OR transness OR transphobia OR transphobic OR transsexual OR transsexualism OR two-spirit OR "women having sex with women" OR "women loving women" OR "women who have sex with women")) OR (SU(2SLGBTQIA OR agender OR asexual OR asexuality OR asexuals OR biphobia OR bisexual OR bisexuality OR bisexuals OR cissexism OR cissexist OR gay OR gayness OR gays OR "gender diverse" OR "gender diversity" OR "gender dysphoria" OR "gender expression*" OR "gender identity" OR "gender minorities" OR "gender minority" OR "gender nonconforming" OR "gender non-conforming" OR "gender queer" OR genderqueer OR "gender transition" OR "gender variant" OR GLBT OR GLBTQ OR heteronormative OR heteronormativity OR homophobia OR homophobic OR homosexual OR homosexuality OR homosexuals OR intersex OR lesbian OR lesbianism OR lesbians OR LGBTQ OR LGBTQIA OR "men having sex with men" OR "men who have sex with men" OR misgender OR misgendered OR misgendering OR nonbinary OR non-binary OR pansexual OR pansexuality OR queer OR queerness OR queers OR "same gender couple" OR "same gender couples" OR "same gender loving" OR "same sex attracted" OR "same sex attraction" OR "same sex couple" OR "same sex couples" OR "sexual and gender minorities" OR "sexual and gender minority" OR "sexual identity" OR "sexual minorities" OR "sexual minority" OR "sexual orientation" OR "sexual preference" OR TGNC OR "third sex" OR "trans female" OR "trans male" OR "trans man" OR "trans men" OR "trans people" OR "trans person" OR "trans woman" OR "trans women" OR transexuality OR transexuals OR transgender OR transgendered OR transness OR transphobia OR transphobic OR transsexual OR transsexualism OR two-spirit OR "women having sex with women" OR "women loving women" OR "women who have sex with women")) | 85,134 |
| 2 | DE "doctoral degrees" OR DE "doctoral programs" OR DE "doctoral students" OR DE "Graduate Education" OR DE "graduate schools" OR DE "Graduate Students" OR DE "Postgraduate students" OR (TI("doctor of philosophy degree" OR "doctoral degree" OR "doctoral degrees" OR "doctoral education" OR "doctoral program" OR "doctoral programs" OR "doctoral student" OR "doctoral students" OR "graduate education" OR "graduate program" OR "graduate programs" OR "graduate school" OR "graduate schools" OR "graduate student" OR "graduate students" OR "graduate study" OR "graduate training" OR "PhD education" OR "PhD program" OR "PhD programs" OR "PhD student" OR "PhD students" OR "PhD training" OR postbaccalaureate OR "Postgraduate students")) OR (AB ("doctor of philosophy degree" OR "doctoral degree" OR "doctoral degrees" OR "doctoral education" OR "doctoral program" OR "doctoral programs" OR "doctoral student" OR "doctoral students" OR "graduate education" OR "graduate program" OR "graduate programs" OR "graduate school" OR "graduate schools" OR "graduate student" OR "graduate students" OR "graduate study" OR "graduate training" OR "PhD education" OR "PhD program" OR "PhD programs" OR "PhD student" OR "PhD students" OR "PhD training" OR postbaccalaureate OR "Postgraduate students")) OR (SU ("doctor of philosophy degree" OR "doctoral degree" OR "doctoral degrees" OR "doctoral education" OR "doctoral program" OR "doctoral programs" OR "doctoral student" OR "doctoral students" OR "graduate education" OR "graduate program" OR "graduate programs" OR "graduate school" OR "graduate schools" OR "graduate student" OR "graduate students" OR "graduate study" OR "graduate training" OR "PhD education" OR "PhD program" OR "PhD programs" OR "PhD student" OR "PhD students" OR "PhD training" OR postbaccalaureate OR "Postgraduate students")) | 39,353 |
| 3 | 1 AND 2 | 712 |

## Academic Search Premier (EBSCOhost)

Search conducted 6/28/23

| **Order** | **Search string** | **Number of results** |
| --- | --- | --- |
| 1 | DE "ASEXUAL people" OR DE "ASEXUALITY (Human sexuality)" OR DE "BIPHOBIA" OR DE "BISEXUALS" OR DE "CROSS-living" OR DE "GAY people" OR DE "GENDER dysphoria" OR DE "GENDER expression" OR DE "gender identity" OR DE "GENDER nonconformity" OR DE "GENDER transition" OR DE "GENDER-nonconforming people" OR DE "HETERONORMATIVITY" OR DE "HOMOPHOBIA" OR DE "homosexuality" OR DE "intersex" OR DE "LESBIANS" OR DE "LGBTQ People" OR DE "LGBTQ+ couples" OR DE "LGBTQ+ identity" OR DE "LGBTQ+ school administrators" OR DE "MALE homosexuality" OR DE "NATIVE American LGBTQ+ people" OR DE "NONBINARY people" OR DE "PANSEXUALITY (Sexual orientation)" OR DE "SEXUAL minorities" OR DE "SEXUAL orientation" OR DE "SEXUAL orientation identity" OR DE "TRANSGENDER people" OR DE "TRANSGENDERISTS" OR DE "TRANSPHOBIA" OR DE "TRANSSEXUALS" OR DE "TWO-spirit people" OR  (TI(2SLGBTQIA OR agender OR asexual OR asexuality OR asexuals OR biphobia OR bisexual OR bisexuality OR bisexuals OR cissexism OR cissexist OR gay OR gayness OR gays OR "gender diverse" OR "gender diversity" OR "gender dysphoria" OR "gender expression*" OR "gender identity" OR "gender minorities" OR "gender minority" OR "gender nonconforming" OR "gender non-conforming" OR "gender queer" OR genderqueer OR "gender transition" OR "gender variant" OR GLBT OR GLBTQ OR heteronormative OR heteronormativity OR homophobia OR homophobic OR homosexual OR homosexuality OR homosexuals OR intersex OR lesbian OR lesbianism OR lesbians OR LGBTQ OR LGBTQIA OR "men having sex with men" OR "men who have sex with men" OR misgender OR misgendered OR misgendering OR nonbinary OR non-binary OR pansexual OR pansexuality OR queer OR queerness OR queers OR "same gender couple" OR "same gender couples" OR "same gender loving" OR "same sex attracted" OR "same sex attraction" OR "same sex couple" OR "same sex couples" OR "sexual and gender minorities" OR "sexual and gender minority" OR "sexual identity" OR "sexual minorities" OR "sexual minority" OR "sexual orientation" OR "sexual preference" OR TGNC OR "third sex" OR "trans female" OR "trans male" OR "trans man" OR "trans men" OR "trans people" OR "trans person" OR "trans woman" OR "trans women" OR transexuality OR transexuals OR transgender OR transgendered OR transness OR transphobia OR transphobic OR transsexual OR transsexualism OR two-spirit OR "women having sex with women" OR "women loving women" OR "women who have sex with women")) OR (AB (2SLGBTQIA OR agender OR asexual OR asexuality OR asexuals OR biphobia OR bisexual OR bisexuality OR bisexuals OR cissexism OR cissexist OR gay OR gayness OR gays OR "gender diverse" OR "gender diversity" OR "gender dysphoria" OR "gender expression*" OR "gender identity" OR "gender minorities" OR "gender minority" OR "gender nonconforming" OR "gender non-conforming" OR "gender queer" OR genderqueer OR "gender transition" OR "gender variant" OR GLBT OR GLBTQ OR heteronormative OR heteronormativity OR homophobia OR homophobic OR homosexual OR homosexuality OR homosexuals OR intersex OR lesbian OR lesbianism OR lesbians OR LGBTQ OR LGBTQIA OR "men having sex with men" OR "men who have sex with men" OR misgender OR misgendered OR misgendering OR nonbinary OR non-binary OR pansexual OR pansexuality OR queer OR queerness OR queers OR "same gender couple" OR "same gender couples" OR "same gender loving" OR "same sex attracted" OR "same sex attraction" OR "same sex couple" OR "same sex couples" OR "sexual and gender minorities" OR "sexual and gender minority" OR "sexual identity" OR "sexual minorities" OR "sexual minority" OR "sexual orientation" OR "sexual preference" OR TGNC OR "third sex" OR "trans female" OR "trans male" OR "trans man" OR "trans men" OR "trans people" OR "trans person" OR "trans woman" OR "trans women" OR transexuality OR transexuals OR transgender OR transgendered OR transness OR transphobia OR transphobic OR transsexual OR transsexualism OR two-spirit OR "women having sex with women" OR "women loving women" OR "women who have sex with women"OR (SU (2SLGBTQIA OR agender OR asexual OR asexuality OR asexuals OR biphobia OR bisexual OR bisexuality OR bisexuals OR cissexism OR cissexist OR gay OR gayness OR gays OR "gender diverse" OR "gender diversity" OR "gender dysphoria" OR "gender expression*" OR "gender identity" OR "gender minorities" OR "gender minority" OR "gender nonconforming" OR "gender non-conforming" OR "gender queer" OR genderqueer OR "gender transition" OR "gender variant" OR GLBT OR GLBTQ OR heteronormative OR heteronormativity OR homophobia OR homophobic OR homosexual OR homosexuality OR homosexuals OR intersex OR lesbian OR lesbianism OR lesbians OR LGBTQ OR LGBTQIA OR "men having sex with men" OR "men who have sex with men" OR misgender OR misgendered OR misgendering OR nonbinary OR non-binary OR pansexual OR pansexuality OR queer OR queerness OR queers OR "same gender couple" OR "same gender couples" OR "same gender loving" OR "same sex attracted" OR "same sex attraction" OR "same sex couple" OR "same sex couples" OR "sexual and gender minorities" OR "sexual and gender minority" OR "sexual identity" OR "sexual minorities" OR "sexual minority" OR "sexual orientation" OR "sexual preference" OR TGNC OR "third sex" OR "trans female" OR "trans male" OR "trans man" OR "trans men" OR "trans people" OR "trans person" OR "trans woman" OR "trans women" OR transexuality OR transexuals OR transgender OR transgendered OR transness OR transphobia OR transphobic OR transsexual OR transsexualism OR two-spirit OR "women having sex with women" OR "women loving women" OR "women who have sex with women")) | 212,047 |
| 2 | DE "DOCTOR of philosophy degree" OR DE "DOCTORAL degree" OR DE "DOCTORAL programs" OR DE "DOCTORAL students" OR DE "GRADUATE education " OR DE "GRADUATE students" OR DE "MINORITY graduate students" OR (TI("doctor of philosophy degree" OR "doctoral degree" OR "doctoral degrees" OR "doctoral education" OR "doctoral program" OR "doctoral programs" OR "doctoral student" OR "doctoral students" OR "graduate education" OR "graduate program" OR "graduate programs" OR "graduate school" OR "graduate schools" OR "graduate student" OR "graduate students" OR "graduate study" OR "graduate training" OR "PhD education" OR "PhD program" OR "PhD programs" OR "PhD student" OR "PhD students" OR "PhD training" OR postbaccalaureate OR "Postgraduate students")) OR (AB ("doctor of philosophy degree" OR "doctoral degree" OR "doctoral degrees" OR "doctoral education" OR "doctoral program" OR "doctoral programs" OR "doctoral student" OR "doctoral students" OR "graduate education" OR "graduate program" OR "graduate programs" OR "graduate school" OR "graduate schools" OR "graduate student" OR "graduate students" OR "graduate study" OR "graduate training" OR "PhD education" OR "PhD program" OR "PhD programs" OR "PhD student" OR "PhD students" OR "PhD training" OR postbaccalaureate OR "Postgraduate students")) OR (SU ("doctor of philosophy degree" OR "doctoral degree" OR "doctoral degrees" OR "doctoral education" OR "doctoral program" OR "doctoral programs" OR "doctoral student" OR "doctoral students" OR "graduate education" OR "graduate program" OR "graduate programs" OR "graduate school" OR "graduate schools" OR "graduate student" OR "graduate students" OR "graduate study" OR "graduate training" OR "PhD education" OR "PhD program" OR "PhD programs" OR "PhD student" OR "PhD students" OR "PhD training" OR postbaccalaureate OR "Postgraduate students")) | 54,272 |
| 3 | 1 AND 2 | 437 |
